# Electrochemical Impedance Spectroscopy-Based Biosensors for Label-Free Detection of Pathogens

**DOI:** 10.3390/bios15070443

**Published:** 2025-07-10

**Authors:** Huaiwei Zhang, Zhuang Sun, Kaiqiang Sun, Quanwang Liu, Wubo Chu, Li Fu, Dan Dai, Zhiqiang Liang, Cheng-Te Lin

**Affiliations:** 1 College of Materials and Environmental Engineering, Hangzhou Dianzi University, Hangzhou 310018, China; zhw5984@hdu.edu.cn (H.Z.); fuli@hdu.edu.cn (L.F.); 2 Qianwan Institute, Ningbo Institute of Materials Technology and Engineering (NlMTE), Chinese Academy of Sciences, Ningbo 315201, China; sunzhuang@nimte.ac.cn (Z.S.); sunkaiqiang@nimte.ac.cn (K.S.); liuquanwang@nimte.ac.cn (Q.L.); chuwubo@nimte.ac.cn (W.C.); 3 State Key Laboratory of Advanced Marine Materials, Ningbo Institute of Materials Technology and Engineering (NIMTE), Chinese Academy of Sciences, Ningbo 315201, China; 4 China College of Chemical Engineering, Zhejiang University of Technology, Hangzhou 310014, China; 5 Faculty of Sports Science, Ningbo University, Ningbo 315211, China; 6 Center of Materials Science and Optoelectronics Engineering, University of Chinese Academy of Sciences, Beijing 100049, China

**Keywords:** biorecognition strategies, nanomaterial enhancement, non-faradaic detection, point-of-care diagnostics, microfluidic integration

## Abstract

The escalating threat of infectious diseases necessitates the development of diagnostic technologies that are not only rapid and sensitive but also deployable at the point of care. Electrochemical impedance spectroscopy (EIS) has emerged as a leading technique for the label-free detection of pathogens, offering a unique combination of sensitivity, non-invasiveness, and adaptability. This review provides a comprehensive overview of the design and application of EIS-based biosensors tailored for pathogen detection, focusing on critical components such as biorecognition elements, electrode materials, nanomaterial integration, and surface immobilization strategies. Special emphasis is placed on the mechanisms of signal generation under Faradaic and non-Faradaic modes and how these underpin performance characteristics such as the limit of detection, specificity, and response time. The application spectrum spans bacterial, viral, fungal, and parasitic pathogens, with case studies highlighting detection in complex matrices such as blood, saliva, food, and environmental water. Furthermore, integration with microfluidics and point-of-care systems is explored as a pathway toward real-world deployment. Emerging strategies for multiplexed detection and the utilization of novel nanomaterials underscore the dynamic evolution of the field. Key challenges—including non-specific binding, matrix effects, the inherently low ΔR_ct_/decade sensitivity of impedance transduction, and long-term stability—are critically evaluated alongside recent breakthroughs. This synthesis aims to support the future development of robust, scalable, and user-friendly EIS-based pathogen biosensors with the potential to transform diagnostics across healthcare, food safety, and environmental monitoring.

## 1. Introduction

Infectious diseases, caused by a diverse array of pathogenic microorganisms including bacteria, viruses, fungi, and parasites, continue to impose a substantial global health and economic burden [1]. The persistent threat from emerging and re-emerging pathogens, dramatically highlighted by recent global health crises such as the COVID-19 pandemic, underscores an urgent and ongoing need for advanced diagnostic technologies [1]. The effective management of infectious diseases, the prevention of widespread outbreaks, and the timely implementation of therapeutic interventions are critically dependent on the availability of rapid, sensitive, specific, and cost-effective detection methods. The capacity for early pathogen identification, even at low concentrations or before the onset of clinical symptoms, can significantly alter disease progression trajectories, inform public health responses, and ultimately save lives. This drives continuous research and development efforts towards novel diagnostic platforms that can overcome the limitations of existing methodologies and meet the evolving challenges posed by infectious agents. The development of such advanced detection systems is not merely an academic pursuit but a societal necessity to bolster global health security and preparedness against future pathogenic threats. The shortcomings of current diagnostic infrastructures, often strained during large-scale outbreaks, further emphasize the necessity for innovative solutions [2]. Traditional diagnostic pathways can be slow, leading to delays in treatment and control measures, which is particularly detrimental in the case of rapidly spreading infectious agents. Consequently, the scientific community is increasingly focused on developing diagnostic tools that are not only accurate but also amenable to deployment in diverse settings, including point-of-care (POC) scenarios and resource-limited environments [3]. This focus is driven by the understanding that accessible and timely diagnostics are a cornerstone of effective infectious disease control [4]. The increasing frequency and impact of global pathogenic threats serve as a powerful catalyst for innovation in this domain, pushing the boundaries of detection technology to provide solutions that are robust, scalable, and adaptable to a wide range of pathogens and epidemiological contexts [5]. Beyond infectious disease diagnostics, EIS is emerging as a versatile bioanalytical engine for non-communicable conditions. Wearable tetrapolar impedance patches have been trialed for real-time cardio-respiratory assessment, while implantable or microfluidic EIS chips have been used to quantify tumor-associated antigens and metabolomic shifts linked to diabetes and cardiovascular disease. Because the technique senses well-defined interfacial parameters—capacitance, charge-transfer resistance and dielectric permittivity—its operation remains physically interpretable, in contrast to opaque algorithm-only approaches [6,7].

Conventional methods for pathogen detection, while often considered gold standards in terms of accuracy, present several inherent limitations that curtail their utility, especially for rapid and on-site applications [8]. Culture-based techniques, which involve isolating and growing microorganisms, are fundamental in microbiology but are notoriously time-consuming, often requiring days or even weeks to yield results [9]. This delay is unacceptable in situations demanding swift intervention, such as acute infections or outbreak investigations [10]. Furthermore, these methods may fail to detect viable but non-culturable (VBNC) pathogens [11] or those with specific growth requirements [12]. Molecular techniques like the polymerase chain reaction (PCR) and its variants, such as reverse transcription-PCR (RT-PCR), offer high sensitivity and specificity for detecting pathogen-specific nucleic acid sequences [13]. However, PCR-based assays typically require sophisticated laboratory equipment and skilled personnel for operation and data interpretation, and involve complex, multi-step sample preparation procedures to extract and purify nucleic acids [14]. These requirements contribute to higher costs and longer turnaround times, limiting their deployment in field settings or resource-constrained laboratories. Moreover, standard PCR detects genetic material, which does not always differentiate between live and dead pathogens, potentially leading to the misinterpretation of active infection. Immunological assays, such as the enzyme-linked immunosorbent assay (ELISA), are widely used for detecting pathogen-specific antigens or host antibodies. While ELISAs can be adapted for high-throughput screening, they often involve multiple incubation and washing steps, extending the assay time. The sensitivity of ELISAs can sometimes be insufficient for detecting low pathogen loads, and the reliance on antibodies can introduce challenges related to antibody production, stability, and cost. The cross-reactivity of antibodies can also lead to false-positive results. Collectively, the time-consuming nature, labor intensity, need for specialized infrastructure and personnel, and often complex sample processing steps of these conventional methods underscore the need for alternative diagnostic approaches that can provide rapid, sensitive, and user-friendly pathogen detection [15]. The progression from these established but often cumbersome techniques towards more agile diagnostic platforms represents a significant shift, driven by the demand for more accessible and timely information in managing infectious diseases [16]. This transition is not merely an incremental improvement but reflects a fundamental rethinking of how diagnostics can be integrated into public health and clinical workflows, especially in scenarios where speed and decentralization are paramount [17].

In response to the limitations of conventional pathogen detection methods, biosensors have emerged as highly promising analytical devices [18]. A biosensor is fundamentally an integrated device that combines a biological recognition element, responsible for selectively interacting with a target analyte, with a physicochemical transducer that converts this biological interaction into a measurable signal [19]. This signal, which can be optical, electrical, mass-based, or thermal, is then processed and displayed, providing quantitative or semi-quantitative information about the presence and concentration of the analyte [20]. Biosensors offer several compelling advantages over traditional techniques, including the potential for rapid response times, high sensitivity enabling the detection of low analyte concentrations [21], excellent selectivity due to the specific nature of the biorecognition event, portability facilitating on-site or POC applications, and often lower costs [22]. Among the various types of biosensors, electrochemical biosensors have garnered significant attention due to their inherent suitability for developing practical diagnostic tools [23]. These devices measure the changes in electrical properties, such as the current, potential, conductance, or impedance, that result from the interaction of the analyte with the biorecognition element immobilized on an electrode surface [24]. Electrochemical biosensors are particularly attractive because of their ease of miniaturization, low power requirements, compatibility with modern microfabrication techniques, and the potential for cost-effective mass production [23].

A significant advancement in biosensor technology is the development of label-free detection strategies [25]. Traditional biosensing often relies on labeling the target analyte or a secondary molecule with reporter tags such as fluorescent dyes, enzymes, or radioactive isotopes to generate a measurable signal. While effective, labeling procedures can be complex, time-consuming, and expensive [26]. They may also interfere with the natural binding affinity of the biomolecules or require specific reagents that are not always stable. Label-free biosensors circumvent these issues by directly measuring an intrinsic property of the analyte or the changes that occur at the transducer surface upon the biorecognition event itself [27]. This direct detection approach simplifies the assay protocol, reduces the time and cost of the assay, minimizes sample handling, and enables the real-time monitoring of binding kinetics. The simplicity and directness of label-free detection make it particularly well-suited for developing rapid and user-friendly diagnostic tools for pathogen detection [28]. The move towards label-free electrochemical biosensors thus represents a convergence of advantageous features, aiming to provide diagnostic solutions that are not only analytically robust but also practically implementable in diverse settings [29].

Among the suite of label-free electrochemical transduction methods, Electrochemical Impedance Spectroscopy (EIS) has distinguished itself as a particularly powerful and versatile technique for biosensing applications, especially for pathogen detection [14]. EIS is a non-destructive analytical method that probes the complex impedance of an electrochemical system by applying a small-amplitude sinusoidal alternating current (AC) voltage or current perturbation over a wide range of frequencies and measuring the corresponding AC current or voltage response [30]. The fundamental strength of EIS lies in its exceptional sensitivity to subtle changes occurring at the electrode–electrolyte interface, where biorecognition events, such as the binding of a pathogen to an immobilized bioreceptor, take place [31]. In the context of label-free biosensing, the binding of target pathogens to bioreceptors immobilized on the electrode surface alters the local electrical properties of this interface. These alterations can manifest as changes in interfacial capacitance, charge transfer resistance, or dielectric characteristics [24]. EIS can precisely measure these impedance changes, which are then correlated with the presence and concentration of the target pathogen. This direct transduction mechanism obviates the need for labeling steps, thereby simplifying the assay design and reducing the potential for interference that labels might introduce [32]. The ability of EIS to provide detailed information about various interfacial processes, including charge transfer, mass transport, and capacitive behavior, makes it an information-rich technique for characterizing biosensor surfaces and monitoring biorecognition events in real-time or near real-time [33]. The versatility of EIS, coupled with its compatibility with various electrode materials and biorecognition elements, has positioned it as a premier choice for developing sensitive and selective label-free biosensors for a wide range of pathogens.

This review aims to provide a comprehensive and critical overview of the advancements in EIS-based biosensors specifically designed for the label-free detection of pathogens. The scope encompasses a thorough examination of the fundamental principles of EIS as they apply to biosensing, detailed design and fabrication strategies, including the selection and immobilization of biorecognition elements and the use of advanced electrode materials and nanomaterials. Furthermore, this review will explore the diverse applications of these biosensors for the detection of various types of pathogens, including bacteria, viruses, fungi, and parasites, with a focus on performance characteristics such as their limit of detection, sensitivity, selectivity, and response time. The primary objectives of this review are threefold: first, it aims to critically analyze the recent progress made in the field over the past five to ten years, with a particular emphasis on the most current breakthroughs and innovative approaches. Second, to identify the existing challenges and knowledge gaps that hinder the widespread practical implementation and commercialization of EIS-based label-free pathogen biosensors. These challenges include issues related to non-specific binding, matrix effects in real samples, sensor stability, reproducibility, and scalability. Third, to offer informed perspectives on future research directions, emerging trends, and the potential impact of these technologies on global health, food safety, environmental monitoring, and biodefense. This review is intended to serve as a valuable and up-to-date resource for researchers, clinicians, engineers, and students working at the interface of electrochemistry, biosensor development, nanotechnology, and infectious disease diagnostics. By consolidating current knowledge and highlighting future opportunities, this review seeks to stimulate further innovation and accelerate the translation of EIS-based label-free pathogen biosensors from laboratory research to practical, impactful applications.

## 2. Principles of EIS for Biosensing Applications

### 2.1. Fundamental Theory of EIS

EIS is a powerful electroanalytical technique that characterizes the frequency-dependent opposition of an electrochemical system to an AC signal [34]. The fundamental principle involves perturbing an electrochemical cell, which. in the context of biosensing. typically comprises a working electrode functionalized with biorecognition elements, a reference electrode, and a counter electrode immersed in an electrolyte solution, with a small amplitude sinusoidal AC potential (E_(t)_ = E_0sin(ωt)_) or current (I_(t)_ = I_0sin(ωt)_) superimposed on a DC potential. Here, E0 and I0 are the amplitudes of the potential and current signals, respectively, and ω is the angular frequency (ω = 2πf, where f is the frequency). The system’s response, a sinusoidal current (I_(t)_ = I_0sin(ωt+ϕ)_) or potential (E_(t)_ = E_0sin(ωt+ϕ)_), is measured. This response will generally be phase-shifted by an angle ϕ relative to the input signal and will have a different amplitude.

The impedance, denoted by Z, is then defined as the ratio of the AC voltage to the AC current as a function of frequency: Z_(ω)_ = E_(ω)_/I_(ω)_. Unlike simple DC resistance described by Ohm’s Law (R = V/I), impedance is a complex quantity because it accounts for both the resistance (energy dissipation) and reactance (energy storage in electric or magnetic fields) of the system [34]. Consequently, impedance is typically expressed as a complex number: Z_(ω)_ = Z′_(ω)_ + jZ″_(ω)_,; here, Z′_(ω)_ is the real (resistive) component and Z″_(ω)_ is the imaginary (reactive) component, and j = −1 [35]. The magnitude of the impedance is given by ∣Z∣ = (Z′)_2_ + (Z″)_2_, and the phase angle is ϕ = arc_tan(Z‴/Z′)_.

The behavior of the electrochemical system can be described using a transfer function, which mathematically relates the output response to the input perturbation in the frequency domain [34]. By sweeping the frequency of the AC signal over a wide range, typically from millihertz (mHz) to megahertz (MHz), EIS can probe various electrochemical and physical processes occurring at different time scales within the biosensor, such as charge transfer kinetics, double-layer charging, and mass transport phenomena [35]. The small amplitude of the perturbation ensures that the system responds linearly, allowing for straightforward mathematical analysis and modeling [36]. This ability to deconvolute complex interfacial phenomena makes EIS a highly informative technique for studying the intricate interactions at biosensor surfaces [37].

### 2.2. Data Representation and Interpretation: Nyquist and Bode Plots

The complex impedance data obtained from EIS measurements are typically visualized using two main types of plots: the Nyquist plot and the Bode plot, each offering unique perspectives on the system’s behavior [35]. The Nyquist plot is a parametric plot of the negative imaginary component of impedance (−Z″) versus the real component (Z′) for each frequency measured [38]. Frequency is an implicit variable in this plot [39]; typically, high-frequency data points appear on the left side of the plot [40], and low-frequency data points appear on the right [41]. A common feature in Nyquist plots for electrochemical systems, including biosensors, is a semicircle in the high to medium frequency range [42]. The diameter of this semicircle is often directly related to the R_ct_ of the electrochemical reaction occurring at the electrode interface [43]. At very high frequencies, the intercept of the plot with the Z′ axis usually represents the R_s_ [44]. At lower frequencies, a linear segment with a slope of 45 degrees (a Warburg element) can appear if the process is limited by the mass diffusion of electroactive species to the electrode surface. Traditionally, Nyquist plots are presented with orthonormal axes (1:1 aspect ratio) to aid in the visual identification of characteristic shapes like semicircles and tilted lines, although modern circuit fitting software has somewhat reduced this necessity [45]. Deviations from perfect semicircles, such as depressed semicircles, often indicate non-ideal capacitive behavior or surface heterogeneity.

The Bode plot consists of two subplots displayed against the logarithm of frequency (log_f_). The first subplot shows the logarithm of the impedance magnitude (log_|Z|_) versus log_f_, and the second shows the phase angle versus log_f_. The Bode magnitude plot provides information about the overall resistance of the system at different frequencies. For instance, at very high frequencies, ∣Z∣ often approaches Rs, while at very low frequencies, it can approach the sum of R_s_ and R_ct_ (and polarization resistance if diffusion is involved). The Bode phase plot is particularly useful for identifying the dominant electrical behavior (resistive, capacitive, or inductive) at specific frequencies. An ideal resistor exhibits a phase angle of 0°, an ideal capacitor −90°, and an ideal inductor +90°. Mixed behavior or non-idealities are indicated by intermediate phase angles [46]. For example, a broad peak in the phase angle plot approaching −90° signifies a dominant capacitive behavior over a certain frequency range, characteristic of the double-layer capacitance [47]. Changes in the shapes, positions, and characteristic values derived from these plots upon the interaction of the analyte with the biosensor surface form the basis of EIS-based sensing [48]. While Nyquist plots are widely used for human interpretation due to their distinct features, recent discussions highlight that alternative data representations—such as impedance magnitude and phase spectra across selected frequency bands—are more amenable to machine learning (ML)-based EIS analysis, which is an emerging area. ML algorithms, particularly supervised classifiers such as support vector machines (SVM), random forests, and convolutional neural networks (CNNs), have been applied to learn complex patterns in high-dimensional EIS datasets, allowing for the accurate detection and differentiation of multiple pathogens from overlapping spectral features. Unsupervised learning approaches (e.g., principal component analysis, t-SNE, and k-means clustering) are increasingly used to identify hidden structures or anomalies in impedance data, aiding exploratory diagnostics and robustness testing. These AI-enhanced frameworks reduce user bias, improve sensitivity, and allow automated decision-making in complex and multiplexed biosensing scenarios. For instance, Buchicchio et al. [49] demonstrated a CNN-based model that achieved over 95% accuracy in classifying EIS responses from different bacteria on the same chip, while Kumar et al. [50] used ML-assisted EIS analysis to simultaneously quantify *E. coli* and *S. aureus* in mixed samples. Integration with microfluidic arrays and edge computing further supports real-time, on-chip data interpretation without reliance on centralized infrastructure, paving the way for fully autonomous, intelligent biosensors.

### 2.3. Equivalent Electrical Circuit (EEC) Models

To quantitatively analyze and interpret EIS data, Equivalent Electrical Circuits (EECs) are employed [34]. An EEC is a theoretical model composed of ideal electrical components such as resistors (R), capacitors (C), and inductors (L) [51], as well as more specialized electrochemical elements like the constant phase element (CPE) and Warburg impedance (Z_W_) [52]. Each element in the circuit is intended to represent a specific physical or electrochemical process occurring at the electrode–electrolyte interface or within the electrochemical cell [53]. By fitting the experimental EIS data (Nyquist or Bode plots) to an appropriate EEC model using complex non-linear least squares (CNLS) fitting algorithms, the values of these circuit elements can be determined, providing quantitative insights into the properties of the biosensor interface [54].

A widely used and fundamental EEC in electrochemical systems is the Randles circuit, which models a simple setup involving a single charge transfer reaction and the semi-infinite diffusion of electroactive species [55]. This circuit typically comprises several key components. The solution resistance (R_s_ or R_sol_) accounts for the resistance of the electrolyte solution between the working and reference electrodes [56]. At the electrode-electrolyte interface, the C_dl_ arises due to the accumulation of ions and oriented dipoles, functioning similarly to a conventional capacitor [57]. The R_ct_ represents the opposition to electron flow during the Faradaic reaction occurring at the electrode surface [58], and it is inversely related to the electrochemical reaction’s rate constant [59]. Additionally, the Z_W_ reflects the impedance resulting from the diffusion of electroactive species to or from the electrode surface [60], becoming particularly significant at low frequencies when mass transport limits the reaction rate [61].

In many real electrochemical systems, particularly those involving modified electrodes or biological interfaces, the capacitive behavior deviates from that of an ideal capacitor [62]. This non-ideality, often attributed to surface roughness, porosity, inhomogeneous current distribution, or specific adsorption, is commonly modeled using a CPE instead of a C_dl_ [63]. The impedance of a CPE is given by Z_CPE_ = 1/(Y_0(jω)_n), where Y_0_ is a proportionality factor (related to capacitance), ω is the angular frequency, and n is an empirical exponent ranging from 0 to 1. If n = 1, the CPE behaves as an ideal capacitor (Y_0_ = C). If n = 0, it behaves as a resistor. If n = 0.5, it can represent a Warburg-like impedance. For depressed semicircles observed in Nyquist plots, n is typically between 0.8 and 0.9.

The evolution of EEC models from simple representations like the Randles circuit to more complex configurations incorporating multiple CPEs, transmission lines for porous electrodes, or elements representing adsorption processes reflects an increasingly sophisticated understanding of the heterogeneous and multi-step nature of biosensor interfaces. This enhanced modeling capability allows for a more accurate deconvolution of various contributions to the overall impedance response, leading to a more nuanced interpretation of the changes induced by biorecognition events. However, it is crucial to select an EEC model that is not only statistically sound in fitting the data but also physically meaningful for the system under investigation. Over-parameterization with unnecessarily complex circuits should be avoided [64].

### 2.4. Mechanism of Signal Generation in Label-Free EIS Biosensors

The core principle of label-free EIS biosensing lies in the ability to detect changes in the interfacial impedance of the sensor electrode upon the specific binding of target pathogens (or their components) to biorecognition elements immobilized on its surface [65]. These binding events alter the local physicochemical environment at the electrode–electrolyte interface, leading to measurable shifts in the impedance parameters derived from EIS data, primarily the R_ct_ and the C_dl_ or CPE parameters [9]. The nature and magnitude of these changes depend on whether the EIS measurement is performed under Faradaic or non-Faradaic conditions [66].

**Figure 1:**
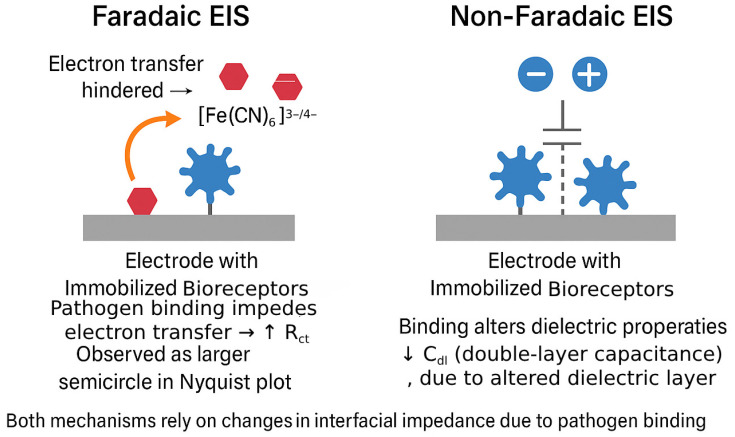
Schematic illustration of signal generation mechanisms in label-free EIS biosensors under Faradaic and non-Faradaic modes. In the Faradaic mode (**left**), pathogen binding hinders the access of the redox probe ([Fe(CN)_6_]^3−^/^4−^) to the electrode surface, thereby increasing the R_ct_. In the non-Faradaic mode (**right**), pathogen attachment alters the dielectric environment at the electrode–electrolyte interface, reducing the C_dl_. Both mechanisms lead to measurable changes in the impedance spectrum.

Faradaic EIS is most often carried out with a freely diffusing redox mediator that shuttles electrons between the working electrode and solution [67]. The anionic ferri/ferrocyanide couple ([Fe(CN)_6_]^3−^/^4−^) is widely used because of its fast outer-sphere electron-transfer kinetics; however, its negative charge can be electrostatically repelled by surfaces that carry net negative charge after biofunctionalization (for example, DNA or sialylated antibodies). In such cases, markedly higher baseline R_ct_ values and compressed signal windows are observed. A convenient remedy is to replace or complement ferri/ferrocyanide with a cationic mediator—most prominently hexaammineruthenium(III/II) chloride ([Ru(NH_3_)_6_]^3+^/^2+^), ferrocenyl-trimethyl-ammonium, or the metallocene methylene-blue cation. Attractive electrostatic interaction lowers the initial R_ct_, while target binding that introduces additional steric hindrance or charge shielding still produces a clear ΔR_ct_. Recent studies with DNA hybridization sensors and IgG immunosensors report up to twofold improvements in sensitivity after substituting RuHex for ferri/ferrocyanide, without compromising linearity or stability [68,69]. Conversely, when the recognition layer is positively charged (e.g., poly-l-lysine or cationic peptides), an anionic probe remains advantageous. This increase in R_ct_ is typically observed as an enlargement of the diameter of the semicircle in the Nyquist plot [70]. The magnitude of this increase in R_ct_ is generally proportional to the amount of pathogen bound to the surface, thus allowing for quantitative detection. In some cases, the binding event might also affect the diffusion of the redox probe, leading to changes in the Z_W_. The distinct mechanisms of signal generation in Faradaic and non-Faradaic EIS biosensors are summarized in Figure 1. In Faradaic systems, electron transfer from a redox probe is modulated by steric and insulating effects introduced by pathogen binding, leading to increased R_ct_. In contrast, non-Faradaic systems respond to changes in the interfacial dielectric environment, manifested as a decrease in C_dl_. These processes enable sensitive detection without the need for external labels, leveraging impedance changes as direct indicators of biorecognition events.

Non-Faradaic EIS, also referred to as capacitive EIS, operates without requiring an external redox probe [14]. In this mode, the biosensor detects variations in the capacitive or dielectric properties at the electrode–electrolyte interface. Since pathogens are biological entities with dielectric properties distinct from those of the surrounding electrolyte or the bioreceptor layer, their binding to the electrode surface induces measurable changes. Specifically, the presence of pathogens can increase the effective thickness of the dielectric layer at the interface, resulting in a reduction in C_dl_ [24]. Additionally, pathogen attachment can alter the surface charge distribution or the permittivity of the interfacial layer, both of which are detectable through EIS. This technique offers a significant advantage in its operational simplicity, as it avoids the complications associated with redox probes, which may interfere with biological components or add complexity to the sensing system.

The choice between Faradaic and non-Faradaic EIS is a strategic one, influenced by the specific characteristics of the pathogen–receptor interaction and the desired sensitivity [71]. Faradaic EIS, by monitoring the easily perturbed electron transfer of a redox probe, can often provide a more pronounced signal change (larger ΔR_ct_), potentially leading to higher sensitivity, especially for detecting subtle binding events or low concentrations of pathogens. Non-Faradaic EIS, while perhaps offering smaller signal changes in some scenarios, directly probes the dielectric changes caused by the pathogen binding and can be simpler to implement. Regardless of the mode, the key is that the biorecognition event induces a quantifiable change in the impedance spectrum, which forms the basis for label-free pathogen detection.

To crystallize the mechanistic distinctions discussed above, Table 1 summarizes the principal advantages and disadvantages of Faradaic and non-Faradaic operation in the context of pathogen biosensing.

Although EIS commonly using the canonical three-electrode cell (working, reference and counter), emerging POC and wearable devices often collapse this architecture to a two-electrode format—typically a micro-fabricated working/pseudo-reference pair printed on the same flexible substrate. This simplification reduces wiring, eases planar layout and halves the front-end electronics, but it imposes three fundamental trade-offs: (i) baseline drift and limited potential control because the ‘reference’ is polarized by the cell current (≈±5 mV h^−1^ in artificial sweat), (ii) convolution of solution resistance (R_s_) with R_ct_, complicating equivalent-circuit fitting, and (iii) reduced accuracy at low frequencies where the double-layer polarization of both electrodes dominates the impedance spectrum. Strategies to mitigate these issues include high-frequency normalization, differential (blank-minus-test) referencing, and migration to tetrapolar (four-terminal) layouts that physically separate current-carrying and sensing electrodes, restoring most of the metrological benefits of the three-electrode cell while remaining skin-compatible. Recent demonstrations illustrate the spectrum of possibilities: a flexible two-electrode sweat patch detected dopamine and glucose with sub-µM resolution [72], while a textile tetrapolar patch captured cardio-respiratory bio-impedance with <2 Ω resolution during treadmill exercise.

## 3. Design and Fabrication Strategies for EIS-Based Label-Free Pathogen Biosensors

The successful development of EIS-based label-free pathogen biosensors hinges on meticulous design and fabrication strategies that encompass the selection of appropriate biorecognition elements (BREs), the choice and modification of electrode materials, and the effective immobilization of BREs onto the transducer surface. Each of these components plays a critical role in determining the overall performance of the biosensor, including its sensitivity, specificity, stability, and reproducibility.

### 3.1. Biorecognition Elements: The Key to Specificity

The specificity of an EIS biosensor is primarily dictated by the BRE, which is responsible for selectively binding to the target pathogen or a unique molecular marker associated with it. A diverse range of BREs have been employed in the development of label-free EIS pathogen biosensors, each with its own set of advantages and limitations.

Antibodies (Abs), including monoclonal (mAbs) and polyclonal (pAbs) antibodies, as well as antibody fragments like Fab (Fragment antigen-binding) and scFv (single-chain variable fragment), are among the most widely used BREs [73] due to their inherent high specificity and affinity for their target antigens [74]. Numerous EIS immunosensors have been developed utilizing antibodies for the detection of various pathogens, such as *Escherichia coli* [14], *Listeria monocytogenes* [75], Influenza A virus (targeting the M1 protein) [76], and SARS-CoV-2 (targeting the N-protein or S-protein) [30]. While offering excellent target recognition, antibodies can be expensive to produce, may suffer from limited stability under harsh environmental conditions, and can exhibit batch-to-batch variability, which can affect sensor reproducibility.

Aptamers are short, single-stranded DNA or RNA oligonucleotides that can fold into specific three-dimensional structures capable of binding to target molecules with high affinity and specificity comparable to antibodies. They are selected through an in vitro process called the systematic evolution of ligands by exponential enrichment (SELEX). Aptamers offer several advantages over antibodies, including ease of chemical synthesis and modification, smaller size, greater stability across a wider range of conditions (temperature, pH), lower production costs, and less immunogenicity. EIS-based aptasensors have been successfully developed for detecting pathogens like *Staphylococcus aureus* [77], *Salmonella typhimurium* [78], Norovirus [78], Avian Influenza H5N1 [78], and SARS-CoV-2 S-RBD protein [79]. The trend towards exploring such synthetic biorecognition elements is driven by the desire for more robust, cost-effective, and customizable sensing platforms.

Bacteriophages (phages) are viruses that specifically infect bacteria, and their inherent host specificity makes them attractive BREs for bacterial detection. Phage-based EIS biosensors can distinguish between viable and non-viable bacterial cells, as phage infection and replication typically occur only in live hosts. They are generally robust and relatively inexpensive to produce. Examples include EIS sensors using phages for *E. coli* and *Listeria innocua*. A notable development is the use of engineered phage receptor binding proteins (RBPs), such as FlaGrab for *Campylobacter jejuni* detection, which can offer tailored binding characteristics and overcome some limitations of using whole phages, like potential cell lysis during detection [80]. However, the stability of immobilized phages, particularly upon drying, can be a concern [81].

Peptides, which are short chains of amino acids, can also serve as BREs [82]. They can be designed or selected to bind specifically to pathogen surface structures [83] or toxins [84]. Synthetic peptides offer advantages such as a small size [85], good stability [86], and easy synthesis and modification [87]. Antimicrobial peptides (AMPs), like magainin I and clavanin A, which interact with bacterial membranes, have been used in EIS biosensors. Synthetic peptides specific to Norovirus have also been employed. While peptides can provide robust recognition, achieving high specificity comparable to antibodies or aptamers can sometimes be challenging [88].

Molecularly imprinted polymers (MIPs) are synthetic polymeric materials with tailor-made recognition sites for a target molecule (template) [15]. They are created by polymerizing functional and cross-linking monomers in the presence of the template pathogen or a characteristic biomarker [89]. After the removal of the template, cavities complementary in size, shape, and chemical functionality to the template are left in the polymer matrix. MIPs offer advantages such as high robustness, stability in harsh chemical and physical conditions, and low production costs. Cell-imprinted polymers (CIPs), a subset of MIPs where whole cells are used as templates, have been applied for the detection of bacteria like *E. coli* and *Staphylococcus epidermidis* [78]. The main challenges with MIPs include achieving uniform binding sites and high specificity, especially for complex analytes like whole pathogens.

**Figure 2:**
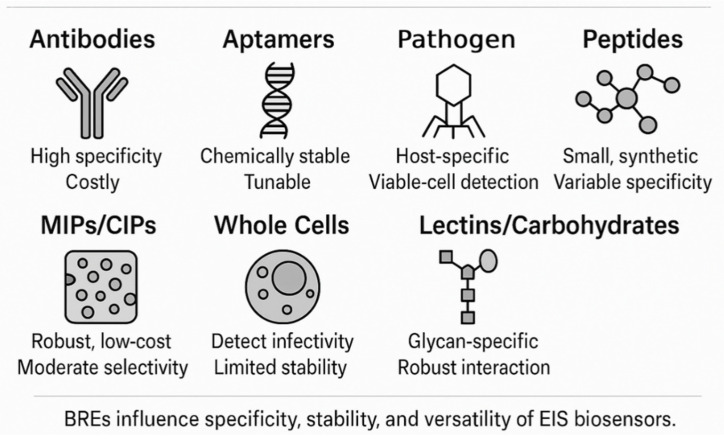
Visual summary of the major types of BREs employed in EIS-based pathogen biosensors. Each class—antibodies, aptamers, phages, peptides, MIPs/CIPs, whole cells, and lectins/carbohydrates—exhibits distinct physicochemical characteristics, advantages, and limitations that influence biosensor specificity, stability, cost, and viability-based detection capacity.

The choice of BRE is a critical design consideration, directly influencing the sensor’s analytical performance [90] and practical applicability [91]. The increasing exploration of synthetic and engineered BREs alongside traditional biological ones reflects a broader movement towards creating more versatile, stable, and cost-effective biosensing systems [92]. Furthermore, the ability of certain BREs, like phages or whole cells, to provide information on pathogen viability is becoming increasingly important for applications where infectivity is a key concern, such as in food safety and clinical diagnostics. Figure 2 provides a comparative visual overview of the principal classes of BREs used in EIS-based pathogen biosensors. These BREs vary widely in their origin (biological or synthetic), molecular recognition mechanisms, and operational robustness. Table 2 and Table 3 summarize the key features, advantages, limitations, and common immobilization strategies of the diverse BREs used in EIS-based pathogen biosensors.

### 3.2. Electrode Materials and Nanomaterial Integration: Enhancing the Transduction Interface

The choice of electrode material and its subsequent modification, particularly with nanomaterials, are pivotal in dictating the sensitivity, stability, and overall performance of EIS-based pathogen biosensors. The electrode serves as the solid support for bioreceptor immobilization [93] and the primary site for electrochemical transduction [94].

Commonly used bulk electrode materials include gold (Au), which is favored for its excellent electrical conductivity, chemical inertness, and well-established surface chemistry, especially for the formation of self-assembled monolayers (SAMs) via thiol-gold bonds, facilitating robust biomolecule immobilization [76]. Carbon-based materials, such as glassy carbon electrodes (GCE), screen-printed carbon electrodes (SPCEs), carbon nanotubes (CNTs), and graphene, offer advantages like low cost, wide electrochemical potential windows, and amenability to various surface modifications [95]. SPCEs, in particular, are well-suited to the mass production of disposable biosensors [96]. Indium tin oxide (ITO) is a transparent conductive oxide often used when optical transparency is required [97], for example, in spectroelectrochemical studies [98] or for certain types of sensor integration [99], though its electrochemical properties can be less ideal than gold or carbon for some applications [100]. Other metals like platinum (Pt) are also employed due to their catalytic properties and conductivity [101].

The integration of nanomaterials has revolutionized electrochemical biosensing by significantly enhancing sensor performance through multiple synergistic mechanisms [21]. Firstly, the inherently high surface-to-volume ratio of nanomaterials substantially increases the effective surface area of the electrode, allowing for a higher loading density of biorecognition elements. This, in turn, boosts the binding capacity and strengthens the overall sensor response. Secondly, nanomaterials such as AuNPs, CNTs, and graphene exhibit exceptional electrical conductivity, which facilitates more efficient electron transfer between the electrode and redox species in Faradaic EIS or improves the conductivity of the sensing layer in general. This enhancement reduces charge-transfer resistance and markedly increases sensitivity. Additionally, certain nanomaterials function as nanozymes, exhibiting enzyme-like catalytic activity [102]. While their direct application in label-free EIS is limited, they can be employed in auxiliary reactions or labeled EIS systems to amplify the signal [103]. Moreover, nanomaterials contribute to improved biocompatibility [104] and provide a stable microenvironment for immobilized bioreceptors, thereby preserving their biological activity and enhancing overall system stability [105].

Specific types of nanomaterials have been extensively employed in EIS-based pathogen sensors due to their unique physicochemical properties. AuNPs are particularly valued for their high conductivity, biocompatibility, and ease of functionalization, especially with thiolated molecules. They serve as conductive bridges and signal enhancers by modifying the electrode surface to increase its area and facilitate electron transfer, as demonstrated in sensors for Hepatitis A Virus, SARS-CoV-2, and HIV-1 DNA. Similarly, AgNPs offer good conductivity along with inherent antimicrobial properties, which can be advantageous in some biosensor configurations. Their primary roles include signal amplification and conductivity enhancement. Magnetic nanoparticles (MNPs), on the other hand, are mainly used for immunomagnetic separation (IMS), enabling the capture and concentration of target pathogens from complex biological matrices before electrochemical detection [106]. This pre-treatment step significantly improves the sensitivity and reduces potential matrix interference.

Carbon-based nanomaterials also play a prominent role in EIS biosensing. CNTs, including single-walled (SWCNTs) and multi-walled (MWCNTs) forms, exhibit outstanding electrical conductivity, mechanical strength, and surface area. These features allow CNTs to form nanostructured electrodes that facilitate faster electron transfer and higher bioreceptor loading, as seen in sensors targeting *Listeria monocytogenes* and *Staphylococcus aureus*. CNT-based electrodes often feature complex 3D architectures that move beyond simple coatings to multifunctional nano-interfaces. Graphene and its derivatives, such as graphene oxide (GO) and reduced graphene oxide (rGO), are also widely applied due to their large theoretical surface area [107], high carrier mobility [108], and functional group versatility [109]. While GO provides oxygenated functionalities suitable for biomolecule immobilization [110], rGO combines conductivity with defect sites that further enhance performance [111]. These materials have been used in EIS biosensors for detecting *S. aureus*, Hepatitis A Virus DNA, and various other pathogens.

In recent years, two-dimensional transition metal materials, particularly transition metal dichalcogenides (TMDs), such as molybdenum disulfide (MoS_2_) and tungsten disulfide (WS_2_), have gained prominence due to their layered structure, tunable surface chemistry, and excellent electrochemical activity. For example, MoS_2_ nanosheets have been utilized to enhance charge transfer kinetics and support dense probe loading in EIS aptasensors, enabling the detection of *Salmonella typhimurium* at femtomolar levels [112]. Similarly, WS_2_ nanostructures have been integrated into immunosensors for bacterial detection with improved impedance signal resolution and reproducibility [113]. These two-dimensional platforms provide versatile and efficient interfaces for EIS biosensor design.

**Figure 3:**
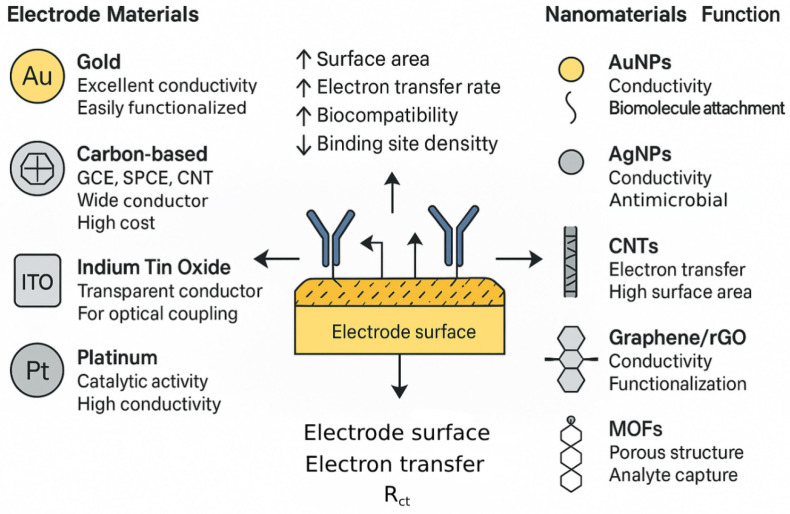
Schematic representation of the integration between electrode materials and nanomaterials in label-free EIS-based pathogen biosensors. Common electrode substrates, such as gold, carbon-based materials, ITO, and platinum, are shown with their key properties. Nanomaterials including AuNPs, AgNPs, CNTs, graphene derivatives, and MOFs enhance sensor performance through an improved surface area, electron transfer, biocompatibility, and functionalization capacity.

To provide a consolidated overview of the nanomaterials discussed above, Table 4 summarizes their key physicochemical properties, functional roles in EIS-based pathogen biosensors, representative examples of pathogen targets, and typical impacts on sensor performance. Beyond traditional nanomaterials, emerging classes such as quantum dots (QDs), metal–organic frameworks (MOFs), and conductive polymers (CPs) are also gaining attention [114]. QDs, though primarily known for fluorescence, have been explored in EIS-based systems due to their unique electronic characteristics [115], typically within hybrid detection schemes [116]. MOFs, with their tunable porosity and high surface areas, serve in diverse roles such as encapsulating bioreceptors, concentrating analytes, or catalyzing electrochemical reactions. MOF-derived materials, especially those converted to conductive porous carbons via pyrolysis, offer improved conductivity and stability, as shown in Cu-MOF/ErGO-based EIS sensors for HBV DNA. Conductive polymers like polyaniline (PANI), polypyrrole (PPy), and PEDOT are easily electrodeposited and function as efficient matrices for bioreceptor immobilization. These materials enhance electron transfer and contribute to signal amplification, with PEDOT-based electrodes being used to detect human influenza A H1N1. To visually consolidate the discussion on electrode and nanomaterial integration, Figure 3 provides a schematic overview of the synergistic interface between the base electrode materials and nanostructured modifiers used in label-free EIS biosensors. The left side of the figure outlines representative bulk electrode materials—gold, carbon-based platforms (such as SPCE and GCE), ITO, and platinum—each offering unique advantages such as high conductivity, chemical stability, optical transparency, or catalytic activity. The central panel illustrates the functional electrode–nanomaterial interface, emphasizing the role of nanomaterials in increasing surface area, enhancing electron transfer rates, improving biocompatibility, and supporting the high-density immobilization of biorecognition elements. On the right, commonly employed nanomaterials are classified by function: gold and silver nanoparticles for conductivity and biomolecular attachment, CNTs and graphene-based materials for high surface area and conductivity, and MOFs for porous architecture and target analyte preconcentration [117]. This integrative interface is essential for achieving high sensitivity and stability in EIS-based biosensing platforms. The synergistic relationship between the bulk electrode material and the chosen nanomaterial modifier is crucial; for example, gold electrodes are readily modified with thiolated nanomaterials, while carbon electrodes often benefit from modification with other carbon allotropes or conductive polymers. This highlights that designing the electrode–nanomaterial system is an integrated process, essential for optimizing sensor performance. In addition, a diverse suite of signal amplification strategies has emerged to overcome the inherently shallow logarithmic dependence of ΔR_ct_ on analyte concentration. Key approaches include (i) nanointerface-driven electron-transfer enhancement using highly conductive architectures such as AuNPs, CNTs, MXenes and 2D-TMDs; (ii) redox-cycling schemes in which mediator molecules shuttle between closely spaced or interdigitated electrodes, yielding >100-fold gains in ΔR_ct_; (iii) catalytic amplification by enzyme labels (e.g., alkaline phosphatase, horseradish peroxidase) whose insoluble or redox-active products magnify impedance shifts; (iv) nucleic-acid cascade reactions such as hybridization–chain reaction, catalytic hairpin assembly and DNAzyme-assisted target recycling that builds high-mass or highly charged networks on the electrode, recently enabling attomolar viral-RNA and microRNA detection; (v) evaporation-enhanced or microfluidic redox-cycling concentrators that physically pre-concentrate analytes while amplifying the electrochemical read-out, achieving the sub-10-particle detection of enveloped viruses within minutes; and (vi) immunomagnetic enrichment, which concentrates bacteria or virions at the sensing interface before impedance measurement. Collectively, these multi-tier strategies can steepen calibration slopes from the customary 10–100 Ω decade^−1^ to >1 kΩ decade^−1^ and push the limits of detection two-to-three orders of magnitude lower while preserving the label-free workflow.

### 3.3. Surface Modification and Bioreceptor Immobilization Techniques: Anchoring Recognition

The method by which biorecognition elements (BREs) are attached to the electrode surface is a critical determinant of an EIS biosensor’s performance, influencing its sensitivity, specificity, stability, and reproducibility [81]. Effective immobilization aims to firmly anchor the BREs while maintaining their biological activity and ensuring optimal orientation for target binding.

SAMs are frequently used to create well-ordered, functionalized surfaces on electrodes, particularly gold [125]. Alkanethiols (e.g., mercaptopropionic acid (MPA), mercaptoundecanoic acid (MUA), cysteamine) spontaneously form dense monolayers on gold via strong sulfur–gold bonds [30]. These SAMs can present terminal functional groups (e.g., -COOH, -NH_2_, -OH) for the subsequent covalent attachment of BREs and can also serve as a barrier to minimize the non-specific adsorption of interfering molecules from the sample matrix [14]. Silanes are similarly used for modifying oxide surfaces like ITO or silicon dioxide. When SAMs are transferred from atomically flat Au (111) to nanostructured substrates (e.g., nanoporous or particulate Au, Au-decorated CNT/graphene, nano-pillars), their long-range order and packing density deteriorate sharply because the local radius of curvature limits van-der-Waals alignment and introduces high step densities. Quantitative X-ray photoelectron spectroscopy and grazing-incidence FT-IR show that ≤70% of alkanethiol coverage is obtained on planar gold when the pore diameter falls below ~50 nm [126], and fluorescence-labelled studies on 15 nm AuNPs report heterogeneous domains with >30° tilt disorder [127]. Electrochemical read-outs corroborate this: incomplete SAMs generate lateral electron ‘leakage’ pathways that flatten the semicircle in Nyquist plots and reduce biosensor-to-biosensor reproducibility [128]. Practical guidelines. (i) Use shorter or branched thiols (C3–C8) and back-filling with hydrophilic spacers (e.g., HS–EG 3) to compensate for curvature-induced steric gaps; (ii) for carbon or MXene nano-topographies, diazonium electrografting or polydopamine priming provide conformal coverage insensitive to roughness and supply -NH_2_/-COOH handles for EDC/NHS coupling [129]; (iii) on nanoporous gold, iterative ligand exchange (‘place-exchange’) combined with electrochemical cycling restores the near-complete SAM order and yields >90% retention of antibody activity after 30 days [126]. Finally, hybrid strategies—SAM formation on the external surface followed by in situ electroless AuNP growth or PEDOT deposition inside pores—have produced uniform, low-impedance interfaces suitable for multiplexed EIS arrays.

Covalent attachment strategies form stable chemical bonds between the BRE and the (modified) electrode surface, leading to robust and durable sensors. A widely used method is carbodiimide chemistry, employing reagents like 1-ethyl-3-(3-dimethylaminopropyl)carbodiimide (EDC) in conjunction with N-hydroxysuccinimide (NHS) to activate carboxyl groups on the surface (or on the BRE) to form amide bonds with amine groups on the BRE (or on the surface) [23]. Glutaraldehyde is another common cross-linker used to link amine groups on the BRE to amine groups on a functionalized surface. For gold electrodes, the direct attachment of thiol-modified BREs (e.g., aptamers, peptides) via Au-S chemisorption is a straightforward covalent method.

Physical adsorption, where BREs are non-covalently adsorbed onto the electrode surface via hydrophobic, electrostatic, or van der Waals interactions, is a simpler approach but generally less reliable. It can lead to random orientation [130], the potential denaturation of the BRE, and the leaching of the BRE from the surface over time [131], resulting in poor sensor stability [132] and reproducibility.

Entrapment in polymers or hydrogels involves physically confining BREs within a polymeric matrix or a hydrogel layer coated on the electrode surface [24]. Conductive polymers can provide an electroactive matrix, while hydrogels offer a hydrated, biocompatible microenvironment that can help preserve the activity of delicate biomolecules like enzymes or antibodies [133].

**Figure 4:**
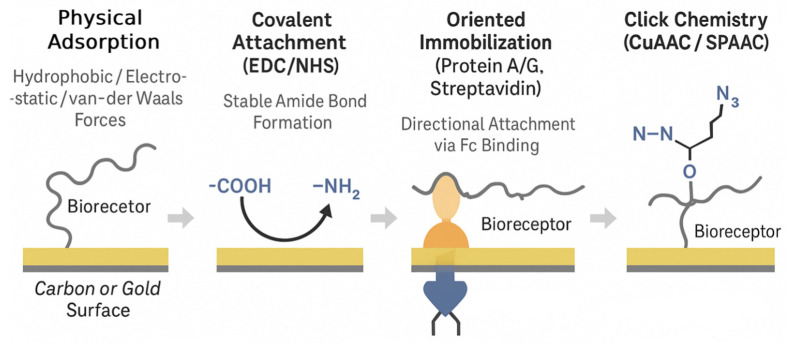
Representative strategies for immobilizing BREs on electrode surfaces in EIS-based biosensors. The illustration compares physical adsorption, covalent attachment via EDC/NHS chemistry, encapsulation in hydrogel or polymer matrices, oriented immobilization (e.g., via Protein A/G or streptavidin-biotin systems), and click chemistry (CuAAC/SPAAC). Each approach differs in stability, orientation control, and compatibility with specific biorecognition elements.

Recognizing the limitations of random immobilization, recent efforts have increasingly emphasized advanced immobilization strategies to achieve greater control over BRE attachment and enhance sensor performance [134]. Among these, oriented immobilization is particularly crucial for antibodies, as it ensures that their antigen-binding sites (Fab regions) remain exposed and accessible to the target pathogen rather than being sterically hindered or oriented toward the electrode surface [135]. Commonly used approaches include the application of Protein A or Protein G, which bind to the Fc region of antibodies, thereby directing the Fab regions outward for optimal target recognition. The biotin–streptavidin system is another widely adopted method, where biotinylated antibodies are bound to streptavidin immobilized on the electrode surface, enabling controlled orientation and high binding efficiency. In addition, click chemistry has emerged as a powerful tool for the precise covalent attachment of BREs. It encompasses highly efficient and bioorthogonal reactions such as the copper(I)-catalyzed azide–alkyne cycloaddition (CuAAC) and the strain-promoted azide–alkyne cycloaddition (SPAAC), which facilitate the conjugation of azide- or alkyne-modified BREs to surfaces functionalized with complementary groups under mild, biocompatible conditions [58]. These reactions form stable triazole linkages with high yield, minimal side reactions, and preserved biomolecular activity, thereby yielding highly stable and reproducible sensor surfaces with well-defined BRE presentation. For instance, click chemistry has been effectively employed to immobilize aptamers on graphene derivatives, leading to enhanced biosensing performance. These immobilization strategies are summarized schematically in Figure 4, which highlights the mechanistic differences and surface interactions underlying each approach.

The progression from simple adsorption to sophisticated, site-specific covalent ligation techniques like click chemistry reflects a deeper understanding of the critical role the bioreceptor interface plays in EIS biosensor functionality. The choice of immobilization strategy is not made in isolation but is closely linked to the nature of the BRE (e.g., antibody vs. aptamer), the electrode material (e.g., gold vs. ITO), and the desired sensor characteristics. Mastering these advanced immobilization techniques is key to developing EIS biosensors with superior sensitivity, specificity, stability, and reproducibility, which are essential for their translation into practical diagnostic tools.

The uniformity and lot-to-lot reproducibility of the sensing interface become paramount once dozens of individually addressed working electrodes must share a single calibration model. The following coating technologies have emerged as front-runners:

Layer-by-layer (LbL) electrostatic assembly—the sequential adsorption of oppositely charged polyelectrolytes/nanomaterials affords Å-level thickness control and coefficient-of-variation (CV) < 3% across wafers up to 100 cm^2^ [136]. LbL is fully aqueous and thus compatible with fragile bioprobes, but its step-wise nature limits high-volume throughput.

Digital inkjet printing—picolitre droplets of graphene, AuNP or Prussian-Blue inks can be gated to defined pixels, enabling bar-coded electrode arrays with inter-spot CV ≤ 5% after coffee-ring suppression using mixed-solvent vehicles [137]. It is entirely mask-less and therefore ideal for the late-stage personalization of multiplex panels.

Aerosol-jet (AJ) and electrohydrodynamic jet printing—sub-10 µm line widths and aspect-ratio-controlled 3D micro-/nano-pillars have recently produced virus-capture impedance patches on polyimide that maintained <4 Ω baseline drift during 60 min of wrist flexion [138]. AJ is additive and compatible with roll-to-roll processing.

Nano-imprint lithography (NIL)—the high-throughput replication (<30 s per wafer) of nano-trenches or nanopillar arrays yields highly uniform “top-down” templates that are subsequently metallized or filled with conductive polymers. Line-edge roughness < 5 nm and sub-2% area-to-area variation have been reported, making NIL attractive for dense impedance microarrays [139].

Polydopamine (PDA)-assisted self-assembly—a one-step dip coating that deposits an ultrathin catecholamine film onto virtually any substrate; subsequent metal ion chelation or click chemistry grafting anchors nanoparticles with RMS roughness < 1.5 nm and batch-to-batch CV ≈ 4% [140]. PDA thereby acts as a universal primer for heterogeneous multiplex panels.

Atomic layer deposition (ALD)—although slower and vacuum-based, ALD provides unsurpassed conformality and sub-nanometer thickness accuracy over complex 3D topologies; it is increasingly used to seed nanocrystal nucleation layers that equalize electrode kinetics across a full array.

Collectively, these approaches offer complementary strengths: LbL and PDA excel in bio-compatibility, inkjet/AJ confer digital patternability, NIL delivers wafer-scale fidelity, and ALD ensures atomic precision. Hybrid workflows—e.g., NIL templating followed by inkjet-printed functional inks—are now entering pilot production for 64-plex impedance chips.

## 4. Performance and Applications of EIS-Based Label-Free Pathogen Biosensors

**Figure 5:**
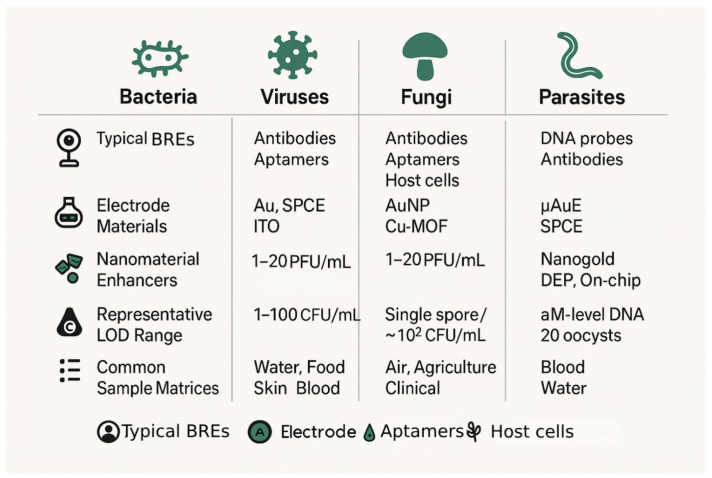
Comparative overview of EIS biosensor design strategies across bacterial, viral, fungal, and parasitic pathogens. Key features include typical biorecognition elements, common electrode materials, enhancement nanomaterials, representative detection limits, and example sample matrices.

Label-free EIS biosensors have demonstrated considerable promise for the detection of a wide spectrum of pathogens, leveraging their ability to transduce binding events at the electrode surface into measurable impedance changes [141]. The performance and applicability of these sensors are often tailored to the specific pathogen type [142], the complexity of the sample matrix, and required analytical parameters such as the detection limits and assay speed [143]. The design of EIS biosensors must be tailored to the unique biological characteristics and diagnostic requirements of different pathogen classes. Figure 5 presents a comparative overview of commonly employed strategies for bacterial, viral, fungal, and parasitic pathogen detection using label-free EIS biosensors.

### 4.1. Detection of Bacterial Pathogens

EIS biosensors have been extensively developed for the detection of various clinically and industrially significant bacterial pathogens. For *Escherichia coli*, a common indicator of fecal contamination and a cause of foodborne illness, numerous EIS-based approaches have been reported. These often utilize antibodies or aptamers as biorecognition elements on gold or carbon-based electrodes. Performance can vary, with limits of detection (LODs) ranging from fewer than 10 colony-forming units per milliliter (CFU/mL) to 10^4^ CFU/mL [14]. For instance, a sensor employing highly conductive tantalum silicide (TaSi_2_) electrodes achieved an LOD of 10^1^ CFU/mL for E. coli O157:H7 in drinking water, while cell-imprinted polymers functionalized on stainless steel microwires detected *E. coli* with an LOD of 2 × 10^2^ CFU/mL. Non-Faradaic EIS has also been applied for *E. coli* detection, with one study reporting the detection of 10 CFU/mL for whole bacteria [14].

*Salmonella* spp., major foodborne pathogens, have also been targeted by EIS biosensors. Antibody- and aptamer-based systems, often enhanced with nanomaterials, have achieved LODs in the low CFU/mL range [15]. The integration of immunomagnetic separation (IMS) with microfluidic EIS platforms has proven effective for *Salmonella* detection in complex food matrices, achieving LODs around 50–70 CFU/mL within an hour [15].

For *Listeria monocytogenes*, a resilient foodborne pathogen capable of growth at refrigeration temperatures, EIS immunosensors and phage-based sensors have shown notable success. The immobilization of anti-*Listeria* monoclonal antibodies on gold electrodes yielded LODs of 4–5 CFU/mL in filtered tomato extract. A biosensor utilizing P100 bacteriophages immobilized on carbon nanotube-modified electrodes demonstrated an LOD of 8.4 CFU/mL for *L. monocytogenes* [119].

*Staphylococcus aureus*, including methicillin-resistant *S. aureus* (MRSA), a significant cause of hospital-acquired and community-acquired infections, has been detected using aptamer- and antibody-based EIS sensors. These often incorporate nanomaterials like CNTs or reduced graphene oxide–gold nanoparticle (rGO-AuNP) composites to enhance sensitivity, with reported LODs as low as 1–10 CFU/mL [14]. A potentiometric aptasensor utilizing SWCNTs detected *S. aureus* in skin models with an LOD of 8 × 10^2^ CFU/mL, illustrating potential for direct clinical sampling [77].

The detection of *Mycobacterium tuberculosis* (Mtb), the causative agent of tuberculosis, or its specific biomarkers is another critical application. EIS immunosensors fabricated on ITO electrodes have been used to detect the CFP10:ESAT6 protein complex, a key Mtb virulence factor, with an LOD of 4.80 ng/mL [144]. Nanomaterial-enhanced genosensors have also been developed for detecting Mtb DNA [123].

*Campylobacter jejuni*, a leading cause of bacterial gastroenteritis, has been targeted by EIS immunosensors employing antibodies or engineered phage proteins. An immunosensor based on O-carboxymethylchitosan surface-modified Fe_3_O_4_ nanoparticles detected *C. jejuni* in a range of 10^3^ to 10^7^ CFU/mL [145]. More recently, a sensor using the FlaGrab phage receptor binding protein immobilized on MWCNT-modified glassy carbon electrodes achieved an LOD of 10^3^ CFU/mL in buffer and an impressive 10^2^ CFU/mL in ex vivo chicken cecal samples, demonstrating utility in complex food-relevant matrices.

Other bacterial pathogens, such as *Vibrio alginolyticus* (LOD 10 CFU/mL using an aptasensor, though potentiometric) [146] and marine pathogenic sulfate-reducing bacteria (SRB) (LODs of 18–21 CFU/mL using anti-SRB antibodies on modified nickel foam or graphene/chitosan electrodes) [78], have also been successfully detected using EIS-based strategies. These examples underscore the versatility of EIS biosensors for a broad range of bacterial targets and sample types.

### 4.2. Detection of Viral Pathogens

EIS-based label-free biosensors have also made significant strides in the detection of various viral pathogens, offering rapid and sensitive alternatives to traditional virological methods.

Influenza virus detection has been a prominent area of research. Immunosensors employing universal anti-M1 antibodies directly attached to gold electrodes have achieved LODs of 20 pg/mL for the M1 protein, corresponding to approximately 80–100 virus particles/µL, with detection times of around 30 min [76]. Aptasensors targeting the influenza A mini-hemagglutinin (HA) protein have reported LODs of 3.7 plaque-forming units (PFU)/mL. Furthermore, hydrogel-based impedance sensors have been developed for Influenza A virus detection, operating in a concentration range of 0.5 to 50 µg/mL and showing promise for air quality monitoring applications.

For human immunodeficiency virus (HIV), EIS biosensors have primarily focused on detecting HIV nucleic acids or related protein biomarkers. An innovative approach utilized a flexible Bi_2_Se_3_ tape electrode modified with AuNPs for the electrochemical sensing of HIV-1 DNA, achieving a very low LOD of 50 amol/L using both DPV and EIS techniques. While the direct EIS detection of HIV proteins is less detailed in the provided materials, the general principles of EIS for protein biomarker detection are applicable [147].

**Figure 6:**
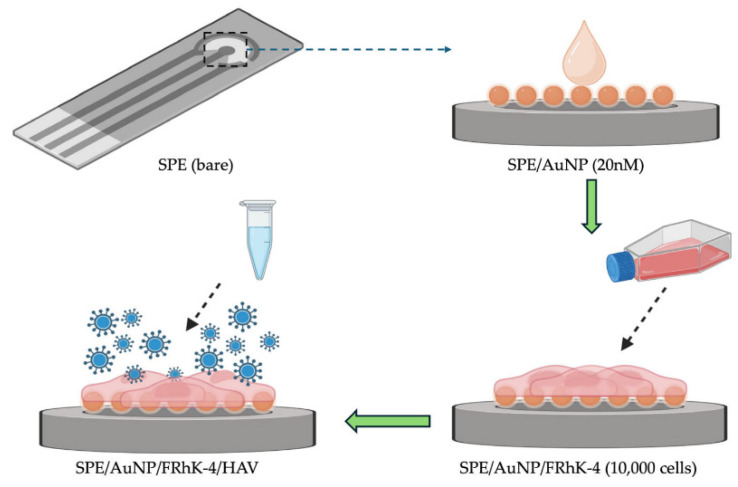
Electrode preparation steps, including the drop-cast deposition of AuNP on bare SPEs, followed by the immobilization of FRhk-4 cells and subsequent testing with HAV [148].

Hepatitis viruses, such as Hepatitis A Virus (HAV) and Hepatitis B Virus (HBV), have been targeted using different EIS strategies. A cell-based EIS biosensor employing FRhK-4 cells (permissive to HAV infection) cultured on AuNP-modified screen-printed electrodes (Figure 6) detected infectious HAV with an LOD of approximately 5 TCID_50_/mL, directly assessing viral infectivity [148]. Label-free electrochemical DNA biosensors have detected HAV cDNA with an LOD of 6.94 fg/µL. For HBV, a sensor based on a Cu-MOF supported on electrochemically reduced graphene oxide (ErGO) has been reported for HBV DNA detection [149].

**Figure 7:**
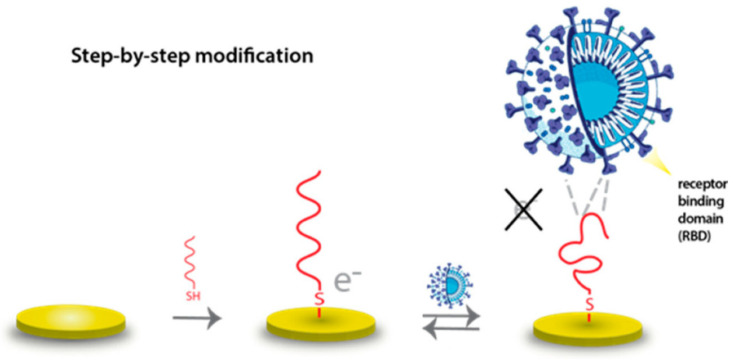
Schematic illustration of one-step electrochemical aptasensor preparation [79].

The recent SARS-CoV-2 pandemic spurred the rapid development of EIS biosensors. Numerous immunosensors targeting viral proteins, such as the nucleocapsid (N) protein or the spike protein’s receptor-binding domain (RBD), have been described. For instance, an EIS immunosensor using gold nanostructured screen-printed carbon electrodes (AuNS/SPCEs) functionalized with anti-N-protein antibodies achieved an LOD of 6 pg/mL for N-protein in saliva samples, with detection possible in under 30 min, including sample processing [30]. Another platform used RBD-coated electrodes for the rapid detection (less than five minutes) of anti-SARS-CoV-2 antibodies in serum [150]. Aptamer-based EIS sensors have also been developed for the SARS-CoV-2 S-RBD protein (Figure 7), with one example showing an LOD of 132 ng/mL [79].

Dengue virus (DENV), a mosquito-borne flavivirus, has been detected using EIS immunosensors. Monoclonal antibodies immobilized on nanostructured alumina/platinum wire electrodes enabled the detection of DENV-2 with an LOD of 1 PFU/mL. The multiplexed detection of DENV-2 and DENV-3 has also been achieved with LODs of 0.23 PFU/mL and 0.71 PFU/mL, respectively, using similar nanostructured electrodes.

Other viruses, including Japanese Encephalitis virus (JEV) (LOD 2 ng/mL using monoclonal antibodies on carbon nanoparticle-modified electrodes) and Norovirus (LODs around 1.7–10 PFU/copies/mL using aptamer or peptide-based bioreceptors on gold electrodes), have also been successfully detected with EIS-based label-free biosensors. These examples highlight the adaptability of EIS for detecting a diverse range of viruses by targeting either viral components or host responses [151].

### 4.3. Detection of Fungal and Parasitic Pathogens

While EIS-based label-free biosensors for bacterial and viral pathogens are more extensively documented, research into their application for fungal and parasitic pathogens is an emerging and important area.

For fungal pathogens, EIS offers a promising avenue for rapid detection, which is crucial given that traditional culture methods for fungi can be particularly slow. Studies have reported EIS biosensors for medically relevant yeasts like *Candida albicans*, often using anti-Candida antibodies on modified electrodes [152]. The detection of *Saccharomyces cerevisiae* has also been investigated using gold-based electrodes with self-assembled monolayers [152]. Furthermore, EIS biosensors have been applied to detect toxigenic fungi in agricultural products, such as *Penicillium sclerotigenum* in infected yams. A notable advancement involves the integration of EIS with microfluidics and dielectrophoresis (DEP) for the capture and quantification of airborne fungal spores, such as *Sclerotinia sclerotiorum*, achieving detection down to the single spore level with rapid measurement times (around 20 s) using aluminum nanoelectrodes [153]. General reviews on biosensors for fungal detection acknowledge the potential of electrochemical methods, including EIS [154].

**Figure 8:**
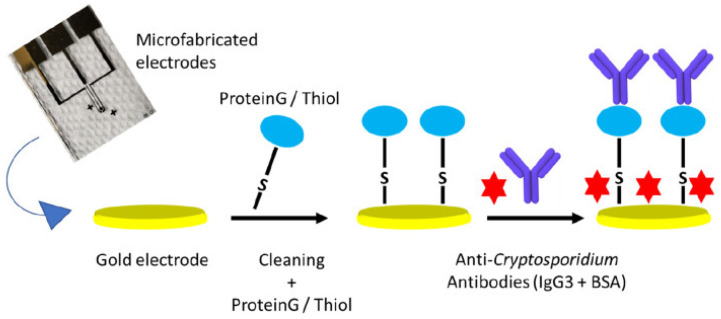
The step-wise process of coating the surface of the microfabricated Au WE with SAM and the immobilization of anti-Cryptosporidium antibodies onto the microfabricated Au WE [141].

The detection of parasitic pathogens using EIS is also gaining traction, particularly for diseases with a significant global health impact. A groundbreaking application is the development of ultrasensitive EIS-based genosensors for malaria diagnosis. These sensors utilize species-specific DNA probes immobilized on micro-gold electrodes (µAuEs) to detect nucleic acids from *Plasmodium falciparum*, *P. malariae*, and *P. ovale* without pre-amplification. Remarkably low LODs in the attomolar (aM) range (e.g., 18.7 aM for *P. falciparum*) have been achieved, with performance in clinical samples showing high sensitivity for purified genomic DNA and promising results for direct detection in whole blood lysates [155]. Another significant development is an on-chip EIS biosensor for the oocysts of *Cryptosporidium*, a waterborne protozoan parasite. This sensor, employing anti-*Cryptosporidium* antibodies on microfabricated gold electrodes (Figure 8), achieved an LOD of approximately 20 oocysts/5 µL, demonstrating potential for water quality monitoring. While the broader application of EIS for other parasitic pathogens, particularly in complex matrices like medical wastewater, is still less explored [156], these examples highlight the capability of EIS to address challenging detection needs in parasitology.

The performance of EIS biosensors is often critically dependent on the sample matrix. While many systems demonstrate excellent LODs in buffered solutions, translating this performance to complex real-world samples such as food homogenates, clinical fluids (blood, saliva, urine), or environmental water remains a significant challenge [30]. Matrix components can cause non-specific binding, electrode fouling, or interfere with the electrochemical measurement itself, often leading to higher LODs or reduced reliability. Addressing this “matrix effect” is a key focus of ongoing research, often involving advanced surface chemistries to resist fouling and integration with sample preparation techniques [157].

Another important consideration is the choice of target analyte—whether to detect the whole pathogen or a specific molecular biomarker (e.g., protein, nucleic acid). Whole pathogen detection can be direct but may face challenges with heterogeneity or require higher concentrations for a detectable signal unless highly efficient capture mechanisms are employed [76]. Biomarker detection, on the other hand, can offer high specificity and potentially lower LODs, especially if the biomarker is abundant or the sensor is exceptionally sensitive to that molecule. However, biomarker detection may necessitate sample processing steps like cell lysis to release the target, which can add to the overall assay time and complexity. The optimal strategy depends heavily on the specific application, the nature of the pathogen, and the characteristics of the sample. To facilitate a comprehensive comparison of recent advances across various pathogen types, Table 5 summarizes the performance metrics of selected label-free EIS biosensors, including their target pathogens, biorecognition elements, electrode modifications, detection limits, assay times, sample matrices, and key references.

### 4.4. Integration with Microfluidics and POC Systems

A significant trend in the development of EIS-based pathogen biosensors is their integration with microfluidic platforms and the drive towards creating portable POC diagnostic devices. Microfluidic systems, often termed “lab-on-a-chip” (LOC) devices, offer numerous advantages for biosensing applications. These include the ability to handle and manipulate minute sample volumes (nanoliters to microliters), which reduces reagent consumption and cost; precise control over fluid flow, enabling automated sample processing steps like mixing, washing, and reagent delivery; and the potential for integrating multiple analytical functions, such as sample pre-treatment (e.g., cell separation, lysis, concentration using techniques like immunomagnetic separation or dielectrophoresis), onto a single miniaturized chip [15]. This integration is crucial for enhancing sensor performance, particularly when dealing with complex biological samples, by minimizing matrix effects and concentrating target analytes at the sensor surface. Examples include microfluidic EIS systems for detecting foodborne bacteria like *Salmonella* [15] and airborne fungal spores [153].

The ultimate goal of such integration is often the development of portable, user-friendly POC diagnostic devices that can provide rapid, on-site pathogen detection without the need for centralized laboratory facilities or highly skilled personnel. EIS is particularly well-suited for POC applications due to its electrical nature, which allows for the straightforward miniaturization of both the sensor and the readout instrumentation (e.g., compact potentiostats). Smartphone integration is also an emerging trend, where the phone’s camera, processing power, and connectivity are leveraged for data acquisition, analysis, the display of results, and even remote data transmission, further enhancing the accessibility and utility of POC biosensors. Despite significant progress, challenges remain in translating these integrated systems into robust, cost-effective, and widely adopted POC tools, particularly concerning issues of sample introduction from real-world sources, long-term stability, and manufacturing scalability. The convergence of EIS with microfluidics and mobile health technologies is paving the way for decentralized diagnostic solutions, which are essential for rapid response during outbreaks and for improving healthcare access in resource-limited settings.

The scale-up of EIS biosensors from proof-of-concept chips to ISO-13485 [160]-compliant products demands concurrent advances in materials processing, electronics integration, regulatory science, and supply chain economics. Recent progress can be mapped onto four mutually reinforcing axes: (i) Mass-manufacturable transducers. Screen-printing, nano-imprint lithography and ink-jet/aerosol-jet techniques already deliver wafer-scale electrode arrays with <5% coefficient-of-variation, while remaining compatible with roll-to-roll polymer substrates. CMOS back-end metallization now routinely embeds thousands of impedance pixels on a single 65 nm die, enabling single-chip, single-use cartridges. (ii) Module-level integration. Battery-free potentiostats and low-drop-out AFE (analog-front-end) ASICs reduce the per-test electronics cost to below USD 1, meeting price targets for wide-area food safety screening. (iii) Quality control and regulatory alignment. The adoption of statistical process control (SPC) for impedance and contact-resistance metrics, coupled with harmonized reference buffers, yielded 95% diagnostic concordance to RT-qPCR in a recent three-lab SARS-CoV-2 ring trial. Guidance such as ISO 23418:2022 [161] (microfluidic IVDs) provides a clear route for dossier assembly. (iv) Market precedents. The Agilent xCELLigence™ platform, now fielded in >2000 pharma labs, demonstrates that impedance hardware and disposable E-plates can be scaled to 50,000 units month^−1^ while maintaining lot-to-lot CV ≤ 3%. Although aimed at cell-based assays, the same supply chain is being repurposed for pathogen cartridges.

### 4.5. Multiplexed Pathogen Detection

The simultaneous detection of multiple pathogens or different strains/biomarkers of a single pathogen from a single sample, known as multiplexed detection, offers significant advantages in diagnostics. It can provide a more comprehensive clinical picture, aid in differential diagnosis, enable rapid screening for multiple contaminants in food or environmental samples, and improve the efficiency of laboratory workflows [162]. EIS-based biosensors are amenable to multiplexing through several strategies.

One common approach involves the use of electrode arrays, where multiple individually addressable electrodes are fabricated on a single chip. Each electrode or a subset of electrodes can be functionalized with a different biorecognition element specific to a particular target pathogen [78]. By monitoring the impedance changes at each electrode, multiple analytes can be detected simultaneously. Another strategy involves creating spatially distinct sensing regions on a single larger electrode, with each region functionalized for a different target.

Peptide-based electrochemical biosensors, including those utilizing EIS, have been highlighted for their potential in developing multiplexed detection systems due to the ease of synthesizing diverse peptide sequences. Membrane-based platforms can also be engineered to incorporate multiple capture zones for different pathogens, with EIS potentially used as the readout mechanism for each zone [163].

However, developing robust and interference-free multiplexed EIS systems presents several challenges. Cross-reactivity between different biorecognition elements or non-specific binding across adjacent sensing sites can lead to false signals. Ensuring uniform fluid delivery and reaction conditions across all sensing elements in an array can also be complex. Furthermore, the instrumentation and data analysis for multiplexed EIS can be more sophisticated than for single-analyte detection. Despite these challenges, the demand for multiplexed diagnostics continues to drive innovation in this area, as it holds the key to more efficient and informative pathogen detection strategies.

## 5. Critical Evaluation, Current Challenges, and Future Perspectives

### 5.1. Critical Evaluation of EIS Technology for Label-Free Pathogen Detection

Electrochemical Impedance Spectroscopy has firmly established itself as a valuable and versatile technique for the development of label-free pathogen biosensors, offering a unique set of advantages. Its label-free nature is a primary asset, as it simplifies assay protocols, reduces the cost and time associated with labeling steps, and avoids the potential interference or alteration of biomolecular interactions that labels might cause. EIS exhibits high sensitivity to minute changes occurring at the electrode–electrolyte interface upon pathogen binding, enabling the detection of low concentrations of analytes [33]. The technique can provide real-time or near real-time data, allowing for the monitoring of binding kinetics and rapid detection [27]. Being a non-destructive method, EIS allows the sample or the sensor surface to be potentially reused or subjected to further analysis [33]. Furthermore, EIS instrumentation and sensor platforms are highly amenable to miniaturization and integration into portable, low-power devices, making them suitable for POC applications [147].

From an analytical standpoint, label-free EIS delivers eight decisive advantages: (i) true label-free operation that shortens assay time and cost; (ii) femto- to attomolar sensitivity arising from its ability to resolve sub-nanometer changes in interfacial capacitance; (iii) real-time kinetic read-out ideal for rapid triage; (iv) non-destructive interrogation that preserves the biorecognition layer; (v) facile microfabrication and low-power electronics that map naturally onto disposable screen-printed formats; (vi) straightforward multiplexing via addressable micro-electrode arrays; (vii) compatibility with microfluidic pre-concentration yielding integrated ‘sample-to-answer’ chips; and (viii) attractive unit economics once produced at scale. These benefits are corroborated by recent systematic reviews and meta-analyses which place impedance platforms among the most cost-effective electrochemical biosensors for POC diagnostics. Nevertheless, six intrinsic constraints continue to impede real-world translation. (1) Non-specific adsorption and matrix effects in protein-rich fluids generate false-positive ΔRct; antifouling zwitterionic monolayers and frequency-gated signal processing reduce this by >80%. (2) Electrode fouling and variability compromise batch-to-batch reproducibility; standardised nanofabrication plus AI-assisted EEC fitting are emerging solutions. (3) Environmental sensitivity (T, pH, ionic strength) necessitates on-chip reference electrodes or ratiometric dual-channel designs. (4) The data interpretation burden demands expertise; cloud-based impedance analytics with embedded equivalent-circuit libraries democratize usage. (5) Regulatory, scaling and cross-laboratory validation hurdles remain; comparative clinical validation against PCR gold standards and the adoption of ISO-13485 manufacturing pipelines will be critical [23]. (6) The logarithmic dependency of R_ct_ on analyte concentration yields calibration curves with inherently shallow slopes (typically 10–100 Ω decade^−1^), so a small experimental error in ΔR_ct_ propagates into a large concentration uncertainty. This fundamental transduction bottleneck helps explain why label-free EIS has not yet achieved routine clinical or industrial uptake. Recent work therefore focuses on redox-cycling nanointerfaces, potentiostatic modulation, and machine-learning-assisted curve fitting to enhance effective sensitivity [34,164]. Recent microfluidic impedance platforms that integrate immunomagnetic separation illustrate how these bottlenecks can be overcome, achieving sub-100 CFU mL^−1^ LODs in milk within 60 min [165].

The complexity of EIS data interpretation can also be a hurdle [35]. Nyquist and Bode plots contain rich information, but extracting meaningful parameters requires careful analysis and the selection of appropriate EEC models. Subtle impedance changes indicative of pathogen binding can sometimes be difficult to discern from instrumental noise, baseline drift, or minor environmental fluctuations. Indeed, EIS measurements can be sensitive to environmental factors such as temperature, pH, and the ionic strength of the measurement solution, necessitating controlled experimental conditions or robust calibration strategies. While EIS is powerful, these limitations highlight the need for careful sensor design, surface engineering, and data analysis protocols to ensure reliable and accurate pathogen detection in practical settings.

### 5.2. Current Challenges in Practical Implementation

Beyond the inherent limitations of the EIS technique itself, several practical challenges hinder the widespread adoption and commercialization of EIS-based label-free pathogen biosensors. Sensor stability and reproducibility are paramount for any reliable diagnostic tool. Achieving long-term operational stability (shelf-life) of the functionalized electrode surface, particularly the activity of the immobilized biorecognition elements, remains a significant hurdle. Bioreceptors can degrade over time or under suboptimal storage conditions. Moreover, ensuring high sensor-to-sensor and batch-to-batch reproducibility in terms of electrode fabrication, surface modification, and bioreceptor immobilization is critical for consistent performance, but can be difficult to achieve, especially with complex nanoscale modifications.

While individual components of EIS biosensors, such as screen-printed electrodes, can be low-cost, the overall cost-effectiveness of the mass production of fully integrated and quality-controlled sensor systems can still be substantial. This includes the costs associated with specialized materials (e.g., highly pure nanomaterials, specific antibodies), precise fabrication processes, and rigorous quality assurance. The scalability of manufacturing processes from laboratory-scale prototypes to high-volume industrial production is another key challenge that needs to be addressed for commercial viability [141].

Commercialization hurdles are multifaceted, encompassing the need for stringent regulatory approvals (e.g., from FDA, EMA), demonstrating clinical utility and cost–benefits compared to established diagnostic methods, and achieving market acceptance among end-users [2]. The path from a promising research prototype to a commercially successful product is often long and resource-intensive.

For POC applications, ensuring true user-friendliness and the seamless integration of all assay steps into a “sample-to-answer” format is crucial. Devices must be operable by personnel with minimal training, and the results should be clear and unambiguous. This often requires sophisticated microfluidic integration for automated sample handling and processing.

A fundamental limitation for some applications is that many EIS biosensors detect the mere presence of a pathogen or its components, without providing information on pathogen viability or infectivity [166]. In contexts like food safety or clinical infection control, knowing whether detected pathogens are alive and capable of causing harm is critical. While some EIS approaches (e.g., cell-based sensors or those using specific phage interactions) can infer viability, this is not a general feature of all EIS biosensors. Addressing these practical challenges is essential for translating the analytical potential of EIS biosensors into tangible benefits for public health and other application areas.

Although more than a thousand proof-of-concept studies now report sub-picomolar limits of detection for pathogen-targeted EIS devices, fewer than a dozen have advanced to formal multi-site validation. Five mutually reinforcing obstacles explain this gap. (i) A lack of certified reference materials and ring-trial protocols: individual groups prepare their own spiked samples, so data cannot be pooled across laboratories, preventing robust meta-analysis [34]. (ii) High inter-platform heterogeneity: variations in electrode micro-architecture, nanomaterial modifiers, biorecognition-layer density and equivalent-circuit fitting mean that the same analyte can yield ΔR_ct_ baselines differing by >25%, making universal cut-off thresholds impossible to define [165]. (iii) Intrinsic logarithmic transfer function: typical calibration slopes are only 10–100 Ω decade^−1^; thus a ±3 Ω thermal or stray-capacitance error propagates into a ±0.3–0.5 log-unit concentration uncertainty, greatly enlarging the cohort size required for statistical power [34]. (iv) Baseline drift and reproducibility limits: electrode fouling and batch-to-batch variation in probe immobilization (>10%) shift baseline impedance by tens of ohms during the weeks or months that validation studies demand, masking pathogen-induced signals [24]. (v) Undefined regulatory pathway and limited funding: unlike glucose meters, no predicate device exists, so developers face de-novo FDA/CE submissions that require costly, multi-center trials typically beyond the scope of academic budgets. Encouragingly, harmonized studies are beginning to break this deadlock: a disposable interdigitated Au/graphite sensor classified 196 saliva specimens for SARS-CoV-2 N-protein with 95% concordance to RT-qPCR, and a microfluidic impedance chip detected Salmonella in pasteurized milk down to 50 CFU mL^−1^ across three laboratories, showing that once common electrodes, buffers and fitting models are standardised, blinded validation is achievable [167].

### 5.3. Recent Breakthroughs and Emerging Trends

**Figure 9:**
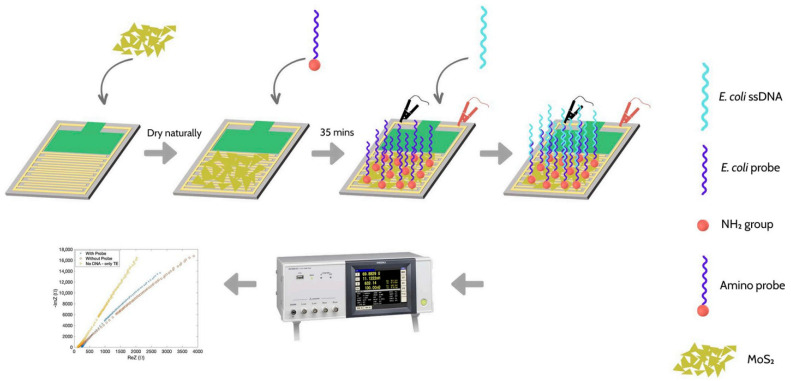
The experimental procedure of *E. coli* DNA detection based on hybrid MoS_2_ nanomaterials IDE sensors [124].

The field of EIS-based label-free pathogen biosensors is dynamic, with continuous innovation aimed at overcoming existing challenges and enhancing performance. Several recent breakthroughs and emerging trends are shaping its future trajectory:

Novel Materials for Enhanced Transduction:

There is a strong impetus towards exploring 2D materials beyond graphene, such as TMDs and MXenes [123]. These materials offer unique electronic, optical, and catalytic properties, large surface areas, and tunable surface functionalities, which can be exploited for creating highly sensitive EIS platforms [168]. For example, MoS_2_ hybrid nanostructures have been used for the ultrasensitive detection of *E. coli* DNA via EIS (Figure 9) [124]. Advanced MOF-derived materials, particularly porous carbons with tailored porosity, high conductivity, and large surface areas, are also gaining attention as robust electrode modifiers or platforms for bioreceptor immobilization. A significant trend is the increasing focus on sustainable and biodegradable materials for biosensor fabrication, such as paper-based electrodes modified with green nanomaterials, and the use of biopolymers, aiming to reduce the environmental footprint of diagnostic devices [169].

Progress in biorecognition includes the development of advanced aptamers through modified SELEX procedures to achieve higher affinity and stability, and the engineering of phage receptor binding proteins or synthetic peptides with tailored specificity and enhanced stability compared to their natural counterparts [80]. A crucial area of advancement is the site-specific and oriented immobilization of bioreceptors. Techniques like click chemistry (e.g., copper-catalyzed azide-alkyne cycloaddition) are being increasingly adopted for their ability to covalently attach bioreceptors to electrode surfaces with high efficiency, specificity, and under mild conditions that preserve biomolecular activity. This leads to more stable and reproducible sensor surfaces with optimally presented bioreceptors, as seen in the oriented immobilization of antibodies for SARS-CoV-2 detection [30].

The complex, multi-frequency data generated by EIS are well-suited for analysis by AI and ML algorithms. ML models are being developed for more accurate pattern recognition in impedance spectra, distinguishing subtle pathogen-induced changes from background noise or drift, classifying different pathogen types or concentrations, and potentially predicting sensor performance or failure. This can lead to improved accuracy, sensitivity, and automation in data interpretation.

Efforts continue to push the limits of detection to extremely low levels. For instance, EIS-based genosensors have demonstrated attomolar sensitivity for detecting Plasmodium DNA without amplification, showcasing the potential for early disease diagnosis [155]. There is also growing interest in wearable and implantable EIS sensors, primarily for the continuous monitoring of physiological analytes, but the underlying principles of miniaturized EIS and biocompatible materials could eventually be adapted for non-invasive or minimally invasive pathogen monitoring in specific contexts.

These breakthroughs collectively indicate a move towards more sophisticated, sensitive, reliable, and user-friendly EIS biosensor systems. The synergy between novel materials, advanced biorecognition strategies, and intelligent data analysis is expected to drive significant improvements in label-free pathogen detection capabilities [170].

### 5.4. Future Outlook and Concluding Remarks

The trajectory of EIS-based label-free pathogen biosensors points towards a future where diagnostics are more accessible, rapid, and integrated into diverse aspects of health and safety management. A primary goal is the realization of true “sample-to-answer” systems, where all steps from sample introduction to result display are automated within a single, often miniaturized, device. This requires sophisticated microfluidic integration for sample preparation (e.g., filtration, concentration, lysis) and precise reagent handling, coupled with robust EIS sensing and automated data interpretation.

Enhanced multiplexing capabilities are highly sought after to enable the simultaneous detection of multiple pathogens, different strains of a pathogen, or associated virulence factors and antimicrobial resistance markers from a single sample. This will be crucial for comprehensive diagnostics, syndromic testing, and efficient screening in food safety and environmental monitoring. Achieving high-density, interference-free multiplexed EIS arrays remains a significant engineering challenge.

A persistent and critical area for future research is improving sensor robustness and reliability in real-world samples. Overcoming matrix effects and non-specific binding is essential for translating laboratory successes into practical field-deployable devices. This will involve continued innovation in surface chemistry (e.g., advanced anti-fouling coatings), novel biorecognition elements with ultra-high specificity, and intelligent sensor designs that can compensate for environmental variations [171].

For widespread clinical and industrial adoption, the rigorous standardization of protocols and comprehensive validation of EIS biosensors against established gold-standard methods are imperative. However, most reported EIS biosensors have not progressed beyond proof-of-concept because five inter-related obstacles hamper rigorous validation: (i) the absence of certified reference materials and harmonized ring-trial protocols; (ii) high inter-platform heterogeneity in electrode micro-architecture, probe chemistry and impedance-fitting models, which prevents direct data pooling; (iii) the logarithmic ΔR_ct vs. concentration relationship (typically 10–100 Ω decade^−1^), whereby small measurement errors propagate into large concentration uncertainties, demanding strict environmental control; (iv) batch-to-batch variability and electrode fouling that erode reproducibility during multi-center studies; and (v) undefined regulatory pathways and limited funding for multi-site clinical or industrial trials. Recent multi-center studies on SARS-CoV-2 N-protein (LOD 6 pg mL^−1^; diagnostic agreement ≥ 95% with RT-qPCR) and Salmonella in milk (LOD ≈ 50 CFU mL^−1^) demonstrate that, once screen-printed gold/graphite electrodes and harmonized buffers are adopted, blinded validation is achievable, underscoring that these barriers are surmountable [172]. This includes establishing clear performance benchmarks, quality control measures in manufacturing, and navigating regulatory approval pathways. The development of reference materials and standardized testing procedures will be vital for ensuring inter-laboratory comparability and building confidence in these emerging technologies.

The focus on sustainability in biosensor development is gaining momentum and will likely become a defining feature of next-generation devices [173]. This encompasses the use of eco-friendly materials, biodegradable substrates, energy-efficient operation, and considerations for the entire lifecycle of the sensor, from manufacturing to disposal.

Ultimately, the aim is to achieve the wider adoption of EIS-based biosensors in resource-limited settings and for decentralized testing. Leveraging low-cost fabrication techniques (e.g., screen-printing, paper-based platforms) and integrating sensors with ubiquitous technologies like smartphones can democratize access to advanced diagnostics, particularly in regions lacking sophisticated laboratory infrastructure. The “critical path” to achieving these practical outcomes involves a concerted, multi-pronged strategy. It is not enough to excel in one aspect, such as achieving ultra-low limits of detection in a laboratory setting; simultaneous advancements are required to improve the core sensing interface (enhancing bioreceptor stability, minimizing non-specific binding, optimizing material conductivity) and in system-level integration (incorporating microfluidics for automated sample preparation, leveraging AI for intelligent data interpretation, and ensuring robust, scalable manufacturing processes). Progress in only one of these areas will be insufficient for broad practical impact.

Furthermore, an interesting dynamic is emerging between the academic pursuit of ultra-high sensitivity (e.g., striving for single-molecule detection capabilities) and the pragmatic need for robust, cost-effective sensors that perform reliably in complex, “dirty” real-world samples. While fundamental research pushing detection limits is vital, for many point-of-care or field applications, a “good enough” limit of detection combined with speed, affordability, and resilience to matrix effects may be more valuable than an exceptionally low LOD that is only achievable under pristine laboratory conditions. This suggests that future developments might see a diversification of EIS platforms, with highly specialized, ultra-sensitive systems for specific research or high-level laboratory applications, and more rugged, cost-effective systems tailored to widespread point-of-need deployment. EIS technology, with its inherent versatility, is well-positioned to serve both these trajectories if designs are appropriately tailored.

In conclusion, Electrochemical Impedance Spectroscopy-based biosensors offer immense potential to revolutionize the label-free detection of pathogens. Their inherent advantages in sensitivity, adaptability, and amenability to miniaturization position them as key enabling technologies for rapid diagnostics in clinical settings, food safety assurance, environmental surveillance, and biodefense. While significant challenges related to real-world applicability, stability, and scalability persist, continuous advancements in materials science, nanotechnology, biorecognition engineering, microfluidics, and data analytics are steadily paving the way for their translation into impactful solutions. Realizing the full potential of these promising technologies will require sustained and collaborative efforts from scientists, engineers, clinicians, and policymakers to bridge the gap between laboratory innovation and practical, globally accessible diagnostic tools that can effectively address the persistent and evolving threats posed by pathogenic microorganisms. The broader concept of “sustainability” will also increasingly shape this field, extending beyond material choices to encompass the energy footprint of devices, the ethical management of diagnostic data, and the overall societal impact of widespread biosensor deployment.

## Figures and Tables

**Table 1:** Comparative analysis of Faradaic and non-Faradaic EIS detection modes.

Attribute	Faradaic EIS	Non-Faradaic (Capacitive) EIS	Implications for Pathogen Biosensors
Signal origin	Change in charge-transfer resistance (ΔR_ct_) caused by hindered electron exchange between electrode and freely diffusing redox probe	Change in double-layer or interfacial capacitance (ΔC_dl_) caused by dielectric/charge redistribution at the electrode surface	Offers complementary transduction pathways targeting either electron-transfer or dielectric perturbations.
Redox probe requirement	Mandatory (e.g., [Fe(CN)_6_]^3−^/^4−^, [Ru(NH_3_)_6_]^3+^/^2+^)	Not required	Eliminating the probe simplifies reagent handling and avoids probe–matrix interactions.
Typical sensitivity (ΔRct or ΔCdl)	Often one to two orders of magnitude larger owing to exponential dependence of R_ct_ on interfacial blockage	Moderate; signal amplitude can be enhanced by nanostructuring or high-κ dielectrics	Faradaic favors ultralow-abundance targets; non-Faradaic can match sensitivity when high-area nanointerfaces are used.
Limit of detection (typical)	10^0^–10^2^ CFU mL^−1^ or sub-pg mL^−1^ proteins in optimized systems	10^1^–10^3^ CFU mL^−1^ or low-ng mL^−1^ proteins (can reach fg mL^−1^ with nano-amplification)	Choice driven by application-specific LOD requirements
Matrix tolerance	Probe diffusion can be hampered by viscous or high-ionic-strength samples; colored matrices may foul electrode	Less affected by bulk diffusion; still sensitive to ionic strength via Debye screening	Non-Faradaic often preferred for turbid food or whole-blood samples
Electrode configuration	Requires stable reference electrode and three-electrode set-up	Two-electrode layouts feasible; reference optional	Non-Faradaic is more amenable to fully printed or disposable chips.
Miniaturization/POC potential	Additional fluidics to refresh redox probe; higher power for stirring/pumping	Simple passive microfluidics; lower power	Non-Faradaic favored in ultra-compact wearables
Susceptibility to surface fouling	High—biofouling blocks electron pathways	Moderate—fouling alters capacitance but may still yield measurable ΔC_dl_	Surface chemistry (e.g., zwitterionic SAMs) critical in both modes
Assay time	Rapid (<5 min) once probe equilibrates	Instantaneous; limited only by binding kinetics	Comparable when identical recognition layers are used
Operational stability	Redox species can degrade (photo-oxidation of ferricyanide)	Excellent shelf-life (no chemical mediator)	Non-Faradaic offers longer storage stability

**Table 2:** Summary of biological bioreceptors used in EIS pathogen biosensors.

Bioreceptor Type	Typical Target(s)	Strengths	Limitations	Recommended Immobilization Chemistry
Antibodies	Bacteria, viruses, toxins	Picomolar affinity; long clinical track-record	Expensive; cold-chain transport; Fc-orientation critical	Protein A/G capture, EDC/NHS coupling
Aptamers	Bacterial cells, viral RNA, toxins	Chemically stable; low cost; PCR-free synthesis	Moderate K_D_; nuclease attack in serum	Thiol–Au SAM, carboxylate–EDC/NHS
Phages	Whole bacteria	Strain-level specificity; self-amplifying	Large biomolecule; limited to bacteria	Cysteine-tag–Au, physical adsorption
Peptides	Bacteria, viruses	Fully synthetic; easy sequence tuning	Lower affinity than antibodies; protease cleavage	Maleimide-thiol, strain-promoted click
Lectins	Viral/bacterial glycans	Recognize glycosylation motifs; inexpensive	Moderate affinity; cross-reactivity	EDC/NHS, diazonium grafting

**Table 3:** Summary of synthetic and cell-based recognition element.

Bioreceptor Type	Typical Target(s)	Strengths	Limitations	Recommended Immobilization Chemistry
Molecularly imprinted polymers (MIPs)	Small molecules, peptides	Solvent/heat-stable, ultra-cheap	Template leakage; size-limited	Electropolymerization on electrode
Cell-imprinted polymers (CIPs)	Whole bacteria, spores	Capture shape & epitope ensemble	Surface roughness may trap debris	Electropolymerization, sol-gel casting
Whole-cell layers	Bacteria, yeast	Natural ligand display; synergistic signal	Viability and biofouling issues	Poly-L-lysine electrostatic deposition
Lectin-functionalized beads	Viral/bacterial glycans	Magnetic separation + recognition	Limited selectivity spectrum	Carbodiimide coupling to beads

**Table 4:** Nanomaterials and advanced materials in EIS-based pathogen biosensors.

Material Type	Key Properties	Role in EIS Biosensor	Examples of Pathogens Detected	Impact on Performance (Typical)	Key References
AuNPs	High conductivity, biocompatibility, ease of functionalization, catalytic activity	Enhance electron transfer, increase surface area for BRE loading, signal amplification, direct pathogen interaction	Bacteria (*E. coli*, *S. aureus*), Viruses (Hepatitis A, SARS-CoV-2, HIV), Mtb	Significant improvement in LOD and sensitivity (e.g., 10–100 fold), faster response	[30]
AgNPs	High conductivity, antimicrobial properties	Enhance conductivity, signal amplification	Bacteria, general pathogen detection	Improved sensitivity, potential for antimicrobial surfaces	[118]
MNPs	Magnetic susceptibility, high surface area	Sample pre-concentration (IMS), separation from matrix, bringing target to sensor surface	Bacteria (*Salmonella*, *E. coli*), Viruses	Indirectly improves LOD by concentrating analyte and reducing matrix effects, enables analysis of larger sample volumes	[15]
CNTs	High surface area, excellent electrical & thermal conductivity, mechanical strength	Enhance electron transfer, increase BRE loading, create 3D electrode architectures	Bacteria (*L. monocytogenes*, *S. aureus*, *E. coli*), Viruses	Lowered Rct, increased capacitive changes, improved LOD and sensitivity	[119]
GO, rGO	Exceptional surface area, high conductivity (graphene), tunable surface chemistry	Enhance electron transfer, platform for BRE immobilization, increase active sites	Bacteria (*S. aureus*), Viruses (Hepatitis A DNA, SARS-CoV-2), Mtb DNA	Dramatically improved sensitivity and LOD (often orders of magnitude), faster kinetics	[120]
QDs	Unique electronic properties, photoluminescence (less relevant for direct EIS)	Can modify electrode conductivity, act as redox mediators or labels in hybrid systems	Viruses (HIV)	Can contribute to signal amplification or unique transduction pathways	[1]
MOFs & Derivatives	High porosity, tunable pore size, large surface area, catalytic sites (MOF-derived carbons: enhanced conductivity)	BRE encapsulation, analyte pre-concentration, catalytic signal enhancement, conductive support	Viruses (HBV DNA)	Improved stability of BREs, enhanced sensitivity due to pre-concentration or catalysis, better conductivity with derivatives	[121]
Conductive Polymers	Intrinsic conductivity, biocompatibility, ease of deposition, porous structure	BRE immobilization matrix, enhance charge transfer, signal amplification, reduce electrode impedance	Bacteria (*E. coli*), Viruses (Influenza A H1N1)	Lowered impedance, enhanced signal changes, improved sensor stability	[122]
MXenes	Large surface area, high electrical conductivity, ease of functionalization	Electrode material, BRE immobilization platform	Mtb (biomarkers)	Potential for high sensitivity and rapid detection	[123]
2D TMDs	Unique electronic properties, layer-dependent bandgap	Sensing layer for DNA hybridization	Bacteria (*E. coli* DNA)	Ultrasensitive detection, potential for specific electronic interactions	[124]

**Table 5:** Performance summary of recent label-free EIS biosensors for pathogen detection.

Pathogen Type	Target Pathogen (Specific)	Biorecognition Element (BRE)	Electrode Material/Modification	Key EIS Parameter Monitored	Limit of Detection (LOD)	Linear/Dynamic Range	Response/Assay Time	Sample Matrix Tested	Redox Probe Used (if Any)	Key Reference(s)
Bacteria	*E. coli* O157:H7	Anti-*E. coli* Abs	TaSi_2_ electrodes	R_ct_	10 CFU/mL	10^1^–10^5^ CFU/mL	<1 h	Drinking water	Not specified	[146]
	*E. coli*	Cell-Imprinted Polymer (CIP)	Stainless steel microwires	R_ct_	2 × 10^2^ CFU/mL	10^2^–10^7^ CFU/mL	30 min incubation	Buffer	Not specified	[158]
	*Salmonella* Typhimurium	Anti-*Salmonella* Abs	PCB IDEs, Magnetic Nanobeads (IMS)	Impedance change	50 CFU/mL	1.8 × 10^3^–1.8 × 10^6^ CFU/mL	60 min	Buffer, Milk	Not specified	[15]
	*Listeria monocytogenes*	Anti-*Listeria* mAb	Au electrode	R_ct_	4 CFU/mL	Not specified	Not specified	Filtered tomato extract	Not specified	[75]
	*Listeria monocytogenes*	P100 Bacteriophage	Quaternized PEI-modified CNTs on electrode	R_ct_	8.4 CFU/mL	10–10^5^ CFU/mL	<1 h	Buffer, Milk	[Fe(CN)_6_]^3−/4−^	[119]
	*Staphylococcus aureus*	Aptamer	rGO-ssDNA-AuNPs composite	Impedance	10 CFU/mL	Not specified	Not specified	Buffer	Not specified	[144]
	*Mycobacterium tuberculosis* (CFP10:ESAT6 protein)	Anti-CFP10 mAb	APTES/ITO electrode	R_ct_	4.80 ng/mL	0.5–50 ng/mL	4 h	Buffer	None (label-free)	[144]
	*Campylobacter jejuni* NCTC 11168	FlaGrab phage protein	MWCNT-GCE, PBSE linker	R_ct_	10^2^ CFU/mL (ex vivo)	10^2^ R_ct_–10^9^ CFU/mL (ex vivo)	~30 min incubation	Chicken cecal samples	[Fe(CN)_6_]^3−/4−^	[80]
Viruses	Influenza A (M1 protein)	Anti-M1 Abs	Au electrode, HDT, GCPs	R_ct_	20 pg/mL (~80–100 viruses/µL)	Not specified	30 min detection	Throat swabs (simulated)	[Fe(CN)_6_]^3−/4−^	[76]
	Influenza A	Antibody	Hydrogel on IDEs	Impedance	0.5 µg/mL (sensitivity 695 Ω·mL/µg)	0.5–50 µg/mL	Not specified	Airborne particles (simulated)	Not specified	[133]
	HIV-1 DNA	DNA probe	Bi_2_Se_3_ tape electrode, AuNPs	R_ct_/DPV signal	50 amol/L	0.1 fmol/L–1 pmol/L	Not specified	Buffer	[Fe(CN)_6_]^3−/4−^	[159]
	Hepatitis A Virus (infectious)	FRhK-4 cells	AuNP-modified SPE	R_ct_	~5 TCID_50_mL	6-log range	6 h incubation	Cell culture medium	[Fe(CN)6]^3−/4−^	[148]
	SARS-CoV-2 (N-protein)	Biotinylated anti-N-protein Ab	SA-BSA/MPA/AuNS/SPCEs	R_ct_	6 pg/mL	0.01–100 ng/mL	<30 min	Saliva (PBS-diluted)	[Fe(CN)_6_]^3−/4−^	[30]
	SARS-CoV-2 (S-RBD)	Thiol-modified Aptamer	Au electrode	R_ct_	132 ng/mL	175 ng/mL–5 µg/mL	2 h prep, fast detection	PBS buffer	[Fe(CN)_6_]^3−/4−^	[79]
Fungi/Parasites	*Sclerotinia sclerotiorum* spores	Dielectrophoresis (DEP) capture	Aluminum nanoelectrodes in microfluidic device	Impedance	Single spore detection	Not specified	~20 s measurement	Air (simulated)	Not specified	[153]
	*Plasmodium falciparum* DNA	Thiol-modified DNA probe	Micro-Au electrodes (µAuEs)	R_ct_	18.7 aM	Attomolar range	< 30 min	Purified gDNA, whole blood lysates	[Fe(CN)_6_]^3−/4−^	[155]
	*Cryptosporidium* oocysts	Anti-*Cryptosporidium* Abs	Protein G/Thiol SAM on microfabricated Au electrode (on-chip)	R_ct_	~20 oocysts/5 µL (4 oocysts/µL)	10–1000 oocysts/5 µL	20 min incubation	Water samples	[Fe(CN)_6_]^3−/4−^	[141]

## Data Availability

No new data were created or analyzed in this study. Data sharing is not applicable to this article.
